# Cancer-intrinsic *Cxcl5* orchestrates a global metabolic reprogramming for resistance to oxidative cell death in 3D

**DOI:** 10.1038/s41418-025-01466-y

**Published:** 2025-03-07

**Authors:** Ramin Seo, Arvie Camille V. de Guzman, Sunghyouk Park, Ji Youn Lee, Suk-Jo Kang

**Affiliations:** 1 Department of Biological Sciences, Korea Advanced Institute of Science and Technology, Daejeon, 34141 Republic of Korea; 2 College of Pharmacy, Natural Product Research Institute, Seoul National University, Seoul, 08826 Republic of Korea; 3 Biometrology Group, Division of Biomedical Metrology, Korea Research Institute of Standards and Science, Daejeon, 34113 Republic of Korea

**Keywords:** Cancer microenvironment, Cancer metabolism

## Abstract

Pancreatic ductal adenocarcinoma is characterized by a three-dimensional (3D) tumor microenvironment devoid of oxygen and nutrients but enriched in extracellular matrix, which acts as a physical and chemical barrier. In 3D, cancer cells reprogram their metabolic pathways in ways that help them survive hostile conditions. However, little is known about the metabolic phenotypes of cancer cells in 3D and the intrinsic cues that modulate them. We found that *Cxcl5* deletion restricted pancreatic tumor growth in a 3D spheroid-in-Matrigel culture system without affecting cancer cell growth in 2D culture. *Cxcl5* deletion impaired 3D-specific global metabolic reprogramming, resistance to hypoxia-induced cell death, and upregulation of *Hif1α* and *Myc*. Overexpression of *Hif1α* and *Myc*, however, effectively restored 3D culture-induced metabolic reconfiguration, growth, redox homeostasis, and mitochondrial function in *Cxcl5*^−/−^ cells, reducing ferroptosis. We also found that pancreatic cancer patients with higher expression of hypoxia and metabolism-related genes whose expression is well-correlated with *CXCL5* generally have poorer prognosis. Together, our findings identify an unanticipated role of *Cxcl5* in orchestrating the cancer metabolic reprogramming in 3D culture that is required for energy and biomass maintenance and that restricts oxidative cell death. Thus, our results provide a rationale for targeting *CXCL5* as a promising therapeutic strategy.

## Facts

- In 3D-cultured cancer cells, *Cxcl5* is required for growth, global metabolic reprogramming, and resistance to hypoxia-induced cell death.
- The metabolic reprograming in 3D culture is secondary to increased expression of *Hif1α* and *Myc*.
- Loss of *Cxcl5* blocked the increase in *Hif1α* and *Myc* expression in 3D-cultured cancer cells.
- PAAD patients expressing higher levels of genes that correlatively are expressed with *Cxcl5* and associated with hypoxia, and metabolism tend toward poorer prognosis.

## Introduction

The tumor microenvironment (TME) comprises not only cancer cells themselves, but also immune cells, fibroblasts, endothelial cells, and pericytes, all of which are critical for regulating cancer initiation and progression [1]. Non-cellular factors in the TME also affect tumor development, such as extracellular matrix (ECM), hypoxia, nutrient availability, pH, and redox status [2]. Certain TME characteristics can present significant barriers to effective cancer treatment by way of immunosuppression, metabolic reprogramming, and physical obstructions to medication transport [2, 3]. Thus, effort toward modifying the TME is required to make cancer more therapy-permissive [1].

Macrophages are one of the most abundant immune cells that infiltrate pancreatic cancer [3] and have an impact on tumor progression and TME remodeling [4]. Although it has been shown that M1, known as classically activated macrophages, have anti-tumoral properties and M2 macrophages promote the cancer progression, the effect of the various macrophage subtypes on tumor development relies on the context and structure of TME and is yet unknown. Therefore, investigating the factors that contribute to macrophages’ context-dependent effect on tumor growth is essential.

Metabolic reprogramming, which is a hallmark of cancer [4, 5], permits cancer growth, proliferation, and survival [6–8]. Cancer metabolism is orchestrated by the cross-talk of cancer intrinsic and extrinsic host factors and systemic metabolism [2]. Although the Warburg effect, a metabolic shift by which tumors produce most of their energy via aerobic glycolysis, has been recognized for a century, tumor metabolic plasticity and the interdependent regulation of tumor metabolism in TME make it challenging to consider the Warburg effect a general feature of all tumors [9]. Nevertheless, many types of tumors show increased glycolytic flux and glycolytic intermediates, indicating a clear role for glycolysis in tumor progression. Notably, aerobic glycolysis not only meets the high energy demands of a growing tumor but also contributes to biomass synthesis and redox balance by feeding into a multitude of pathways, including the pentose phosphate pathway (PPP), the hexosamine pathway, de novo serine/glycine synthesis, and one-carbon metabolism [7, 10, 11].

One-carbon metabolism comprises a linked set of metabolic processes, including the folate and methionine cycles, which play critical roles in biomass (nucleotides, protein, and lipids) synthesis, methylation, and redox homeostasis. Previous studies have demonstrated that certain types of cancer show increased de novo synthesis of serine, which is formed from the glycolytic intermediate 3-phosphoglycerate (3-PG) through a series of reactions mediated by phosphoglycerate dehydrogenase (PHGDH), phosphoserine aminotransferase 1 (PSAT1), and phosphoserine phosphatase (PSPH). Serine hydroxymethyltransferase 1 or 2 (SHMT1/2) can convert serine to glycine, providing single carbon units to the folate cycle. A trans-sulfuration process in the methionine cycle conjugates homocysteine and serine, producing cystathionine, and then cysteine. Through glutamate cysteine ligase (GCL) and GSH synthetase (GSS), glycine is connected to cysteine and glutamate to produce glutathione (GSH) [10, 12]. GSH is responsible for maintaining redox balance in combination with reduced nicotinamide adenine dinucleotide phosphate (NADPH) produced by the folate cycle and PPP. Tumors are known to increase serine/glycine production to preserve redox homeostasis in the hypoxic TME, where the supply of serine and glycine is minimal [13]. Therefore, aerobic glycolysis is essential for providing the building blocks and antioxidants necessary to sustain cancer proliferation and survival.

Although many studies have revealed that cell-ECM interactions and cell-cell interactions can alter cell proliferation, migration, and metabolism in cancer cells [14, 15], 2D monolayer cultures are severely limited in their ability to recapitulate the interactions that exist in 3D TME. Importantly, although a few publications have documented metabolic modifications that occur in a 3D environment [16], we still lack a comprehensive view of the metabolic landscape of cancers in the 3D TME and the cancer-intrinsic cues that confer 3D-specific metabolic reprogramming. To identify such cancer-derived factors, we developed a reproducible and controlled cancer spheroid culture model to mimic the 3D TME where cancer cells interact with surrounding cells and the ECM and respond to non-cellular characteristics including hypoxia. Using this culture system, we discovered an unanticipated role for cancer-derived *Cxcl5* in the metabolic reprogramming of cancer cells as they adapt to the 3D TME.

## Results

### Cancer-derived CXCL5 is necessary for tumor spheroid growth

**Fig. 1:**
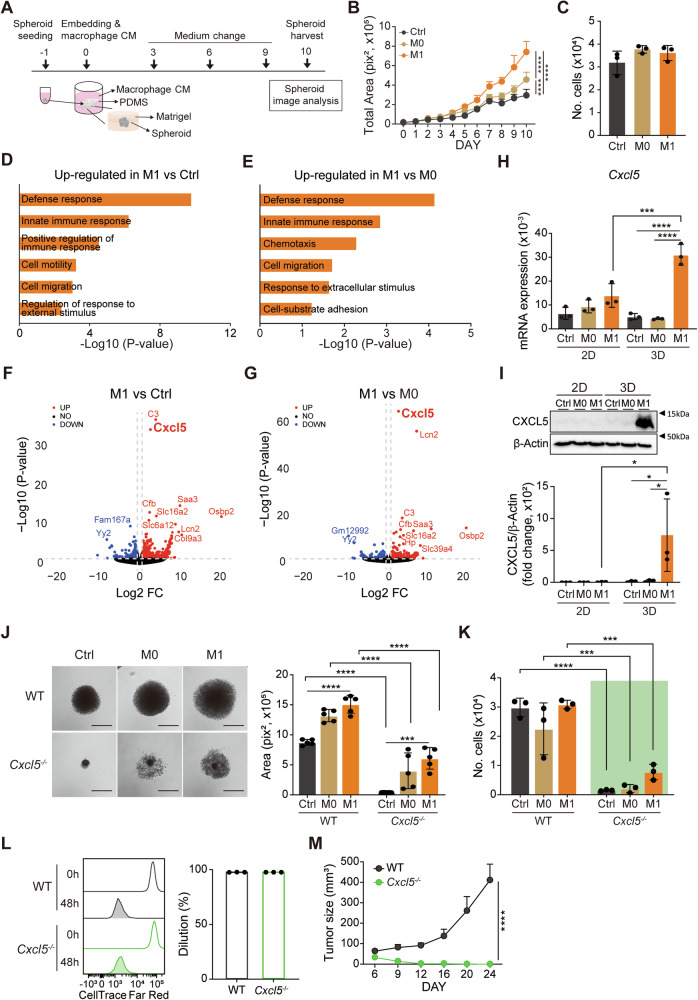
M1-CM treatment-induced upregulation of CXCL5 expression is necessary for tumor spheroid growth. **A** Schematic showing the 3D spheroid-in-Matrigel culture system. Panc02 spheroid-in-Matrigel were cultured without (Ctrl, control) and with M0- or M1-CM supplementation before being harvested on day 10. **B** Graph comparing total area of spheroids (*n* = 5 spheroids/group). **C** The number of cells in each spheroid was measured via CyQUANT^TM^ assays. (*n* = 3). **D, E** GO analysis of differentially expressed genes (DEGs) in the comparison of Control (Ctrl) and M1-CM-treated Panc02 spheroids **(D)** or the comparison of M0-CM and M1-CM-treated Panc02 spheroids (**E)**. **F, G **Volcano plots showing DEGs in the comparison of M1-CM-treated spheroids and control (Ctrl, **F**) or M0-CM-treated spheroids (**G)**. **H**, **I** *Cxcl5* expression in 2D- or 3D-cultured Panc02 with or without macrophage CM (*n* = 9–12 spheroids/group), as analyzed by qRT-PCR (**H)** and western blotting (**I**, upper). Bar graph showing the quantitation of CXCL5 expression, normalized to the amount of β-actin (**I**, bottom). **J** Bright field spheroid micrographs (left) and bar graph showing the total area of spheroids (right) (*n* = 5 spheroids/group). Magnification, 4X. Scale bar = 1 mm. **K** Number of cells per Panc02 spheroid (*n* = 3 spheroids/group). **L** Flow cytometry plots (left) showing CellTrace^TM^ dye dilutions and bar graphs (right) showing dilution percentages (MFI difference between 0 h and 48 h of WT cells was set to 100%). Significance was assessed via unpaired two-tailed Student’s *t*-tests. **M** Tumor growth of WT and *Cxcl5*^−/−^ Panc02 cells subcutaneously injected into mice (*n* = 4 mice/group). **B,** **C,** **H**–**M** Data are representative of three independent experiments (*n*, number of spheroids/group) and presented as mean ± SEM. Significance was assessed via two-way (**B,** **H,** **I,** **J,** **K,** **M)** or one-way (**C)** ANOVAs with Tukey’s post-hoc tests.

Pancreatic cancer is typically accompanied by TME macrophage infiltration and desmoplasia [17]. While tumor-infiltrating macrophages help shape an immunosuppressive TME [18], ECM desmoplasia modulates both the behavior of cancer cells and tumor-infiltrating macrophages [19]. To recapitulate the macrophage-conditioned 3D characteristics of the TME, we established a 3D tumor spheroid-in-Matrigel culture system using the murine pancreatic (Panc02) and colon (MC38) adenocarcinoma cell lines and treated macrophage conditioned media obtained from M0 (untreated) or M1 (IFNγ plus lipopolysaccharide-treated) macrophages (Fig. 1A, Supplementary Fig. 1A, B). We found that M1-CM treatment dramatically increased total Panc02 spheroid area (Fig. 1B, Supplementary Fig. 1C) without increasing cell proliferation (Fig. 1C), resulting in the reduction of spheroid compactness (Supplementary Fig. 1D). The size increase by M1-CM treatment was also observed in MC38 spheroid (Supplementary Fig. 1E). In a Gene Ontology (GO) analysis of the differentially expressed genes (DEGs, Table S2) following RNA sequencing, we found M1-CM increased the expression of genes associated with innate immune responses, chemotaxis/migration compared to control or M0-CM (Fig. 1D, E). Indeed, the migration of cells from the M1-CM treated spheroids was enhanced compared to control or M0-CM treatment (Supplementary Fig. 1F). Additionally, we found increased expression of several genes associated with epithelial-mesenchymal transition (EMT) [20] (Supplementary Fig. 1G). Among the genes upregulated by M1 treatment, the increase in expression for the chemokine *Cxcl5* was notably large (Fig. 1F, G). Additionally, using western blotting, we verified that CXCL5 expression was elevated in 3D culture compared to 2D culture (Supplementary Fig. 1H) and M1-CM treatment significantly upregulated CXCL5 expression exclusively in 3D culture, not 2D culture (Fig. 1H, I).

To see whether similar results are found in M1 macrophages polarized by stimuli other than IFNγ + LPS and M2 macrophages by IL-4 or TGF-β1, we stimulated the macrophages with a tumor conditioned media of 2D- (2D TCM) or 3D-cultured tumor cells (3D TCM), IFNγ alone, TNFα, IL-4 or TGF-β1. Additionally, we included an IFNγ + TNFα co-stimulation condition as IFNγ has been shown to prime macrophages, leading to boosting up their responsiveness to inflammatory molecules, such as TNFα [21]. We first verified that the IFNγ addition to the stimulation (IFNγ alone, IFNγ + TNFα, or M1) induced the upregulation of CD86 and MHC class II molecules and the expression of iNOS (*Nos2*), the markers for classically activated macrophages (Supplementary Fig. 1A, B). These conditions did not upregulate the expression of CD206, an M2 marker [22]. Meanwhile, IL-4, but not TGF-β1, treatment increased expression of CD206 (Supplementary Fig. 1A). We then treated the 3D tumor cells with the conditioned media from these activated macrophages and investigated 3D tumor growth and CXCL5 expression. We confirmed that the 3D growth of tumor cells and the expression of CXCL5 were increased by the conditioned media from IFNγ + TNFα co-treated macrophages, similar to those of IFNγ + LPS stimulation (Supplementary Fig. 1I, J). Interestingly, although the status of macrophage and CXCL5 expression was not changed by TGF-β1 stimulation, the conditioned media of TGF-β1-treated macrophages enhanced the 3D tumor growth. Altogether, our results suggest that IFNγ is critical for promoting the growth and the expression of CXCL5 of 3D tumor cells and that TNFα may be a downstream effector molecule of LPS-stimulation.

Furthermore, our in vitro results are corroborated by our analysis using the Tumor Immune Estimation Resource (TIMER) 2.0 database, which shows a significant correlation between CXCL5 expression and M1 macrophage infiltration in numerous cancer types (Supplementary Fig. 1K). Collectively, tumor spheroids increased in size along with increase of CXCL5 expression in response to M1 macrophage-conditioned media.

To study the role of cancer-derived CXCL5 in the growth of M1-CM-treated spheroids, we deleted *Cxcl5* in Panc02 cells using CRISPR/Cas9 (Supplementary Fig. 2A) and performed a 3D spheroid-in-Matrigel culture of wild-type (WT) or *Cxcl5*^−/−^ Panc02 cells with or without M0- or M1-CM. Unexpectedly, we found *Cxcl5* deletion reduced spheroid growth with fewer cells in *Cxcl5*^−/−^ than WT spheroids in all conditions (Fig. 1J, K). We detected that 3D culture significantly increased cell death assessed by annexin V staining (Supplementary Fig. 2B). Although, M1-CM treatment reduced the 3D culture-associated cell death (Supplementary Fig. 2C), the M1-CM is not sufficient to fully overcome the intrinsic defect by *Cxcl5* deficiency (Fig. 1J). There was no difference, however, between the proliferation rates of 2D-cultured WT and *Cxcl5*^−/−^ cells (Fig. 1L). These data suggest that CXCL5 is a 3D-dependent requisite for tumor survival and/or growth. Indeed, we found that treatment of Panc02 cells with recombinant CXCL5 (rCXCL5) did not alter 2D proliferation rate while it increased the size of the individual cells (Supplementary Fig. 2D, E). Meanwhile, rCXCL5 increased the size and cell numbers of spheroids with no change in cell size of individual cells in the spheroids (Supplementary Fig. 2F–H). Additionally, *Cxcl5* complementation in *Cxcl5*^−/−^ Panc02 cells restored the growth retardation of *Cxcl5*^−/−^ spheroids (Supplementary Fig. 2I). In corroboration of this result, we found reduced in vivo tumor growth of *Cxcl5*^−/−^ Panc02 cells compared to WT after subcutaneous injection into mice (Fig. 1M).

Furthermore, CXCL5 was found to have a similar effect on 3D tumor growth in the mouse colon cancer cell line MC38, the human pancreatic cancer cell lines MIA PaCa-2 and PANC-1, and the human lung cancer cell line A549 (Supplementary Fig. 2J). Together, our results indicate that 3D condition, but not 2D condition, elicits the cancer-intrinsic role of *Cxcl5* for the growth of a variety of mouse and human cancers.

### *Cxcl5* deletion impaired global metabolic reprogramming in 3D

**Fig. 2:**
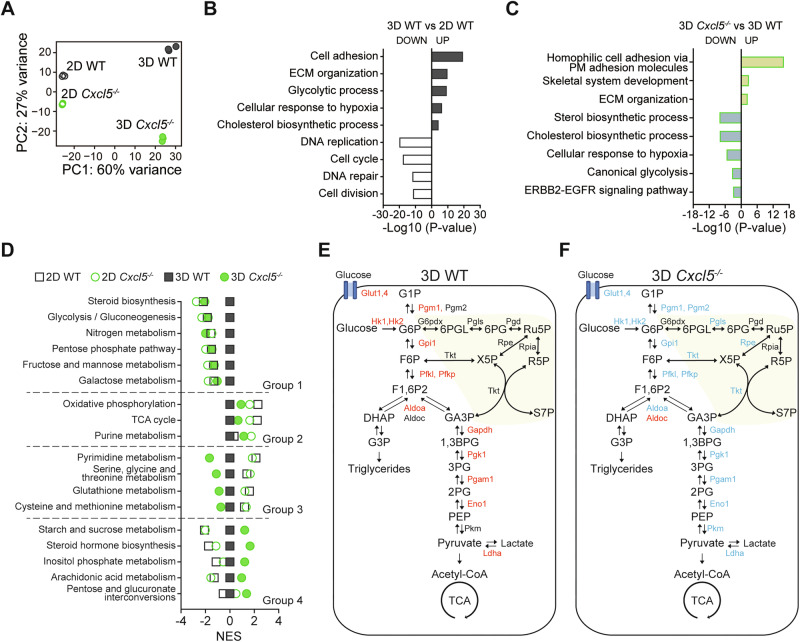
*Cxcl5* deficiency impairs metabolic reprogramming in 3D-cultured tumor spheroids. **A** PCA analysis of 2D- or 3D-cultured WT and *Cxcl5*^−/−^ Panc02 cells. **B**, **C** GO analysis of DEGs in the comparison between 3D- and 2D-cultured WT Panc02 cells (**B)** or between 3D-cultured *Cxcl5*^−/−^ and WT Panc02 cells (**C)**. **D** GSEA analysis of metabolic pathway genes. **E**, **F** Schematic of the glycolysis-related pathways showing DEGs in the comparison of 3D-cultured to 2D-cultured WT cells (**E)** or 3D-cultured *Cxcl5*^−/−^ to 3D-cultured WT cells (**F)**. Up- and down-regulated genes appear in red and blue, respectively. The pentose phosphate pathway appears in yellow. G1P Glucose 1-phosphate, G6P Glucose 6-phosphate, F6P Fructose 6-phosphate, F1,6P2 Fructose 1, 6-bisphosphate, GA3P Glyceraldehyde 3-phosphate, 1,3BPG 1,3-bisphosphoglycerate, 3PG 3-phosphoglycerate, 2PG 2-phosphoglycerate, PEP Phosphoenolpyruvate, DHAP Dihydroxyacetone phosphate, G3P Glycerol 3-phosphate, 6PGL 6-phospho-gluconolactone, 6PG 6-phospho-gluconate, Ru5P Ribulose 5-phosphate, R5P Ribose 5-phosphate, X5P Xylulose 5-phosphate, S7P Sedoheptulose 7-phosphate.

To better understand the cause of the growth defect in *Cxcl5*^−/−^ cells in 3D, we performed an RNA sequencing. A principal component analysis (PCA) showed similar gene expression profiles between WT and *Cxcl5*^−/−^ cells in 2D, but distinct in 3D (Fig. 2A). This suggests cancer cells in 3D conditions require *Cxcl5*-dependent gene expression to grow and survive. In a GO analysis, we found higher expression of genes associated with cell adhesion, ECM organization, and hypoxic responses in 3D than 2D conditions. In addition, we found an upregulation of genes regulating metabolic processes such as glycolysis (*Aldoa, Pgk1, Ldha*) and cholesterol synthesis (*Sqle, Msmo1, Dhcr7*) in 3D WT compared with 2D WT cells. In contrast, we found reduced expression of genes associated with DNA replication (*Fen1*, *Mcm7*, *Gins1*), cell cycle (*Cdk1, Ccnb1, Cdc6*) and DNA repair (*Pcna, Rnf8, Cgas, Traip*) in 3D- than 2D-cultured WT cells (Fig. 2B). Remarkably, we found that loss of *Cxcl5* reversed the increased expression of the genes associated with hypoxic responses, glycolysis, and cholesterol synthesis (Fig. 2C). These results indicate *Cxcl5* plays a critical role in cancer cells adapting their metabolism and hypoxic responses to 3D culture environment.

Next, we conducted a Gene Set Enrichment Analysis (GSEA) analysis of metabolic pathways using the Kyoto Encyclopedia of Genes and Genomes (KEGG) database. We found 2D- or 3D-cultured WT and *Cxcl5*^−/−^ cells showed significant differences in the expression of genes associated with 18 metabolic pathways. We clustered these metabolic pathways into 4 groups based on their expression patterns when compared to 3D-cultured WT cells (Fig. 2D). Group 1 represents the genes of various metabolic pathways upregulated in 3D spheroid culture versus 2D culture but not in 3D-cultured *Cxcl5*^−/−^ cells. These pathways include not only glycolysis and the pentose phosphate pathway but also steroid biosynthesis, nitrogen metabolism, and monosaccharide (fructose, mannose, and galactose) metabolism. Indeed, expression of glycolytic enzymes were significantly upregulated in 3D-cultured cells but not in *Cxcl5*-deficient cells (Fig. 2E, F). Group 2 included genes decreased in 3D culture compared to 2D culture but increased slightly in 3D-cultured *Cxcl5*^−/−^ cells. Expression levels of genes associated with oxidative phosphorylation and the tricarboxylic acid (TCA) cycle were reduced in 3D culture compared to 2D culture. This is consistent with cancers exhibiting low levels of oxidative phosphorylation and high levels of glycolysis [5, 7], suggesting that our spheroid-in-Matrigel culture faithfully recapitulated the metabolic reprogramming of the TME. Group 3 contained genes decreased in 3D culture compared to 2D culture and further decreased in 3D-cultured *Cxcl5*^−/−^ cells. Group 3 genes include metabolic genes associated with one-carbon metabolism (serine/glycine metabolism, cysteine and methionine metabolism, and glutathione metabolism). Importantly, we found 3D-cultured *Cxcl5*^−/−^ cells showed reduced expression of both group 1 and group 3 genes compared to WT cells (Fig. 2D). Group 4 included genes increased in 3D-cultured WT and further increased in 3D-cultured *Cxcl5*^−/−^ cells. Collectively, the results indicate that loss of *Cxcl5* impedes the global reprogramming of metabolic pathways critical for cancer growth in a 3D environment.

### Cancer cells lacking *Cxcl5* are more sensitive to hypoxic cell death

**Fig. 3:**
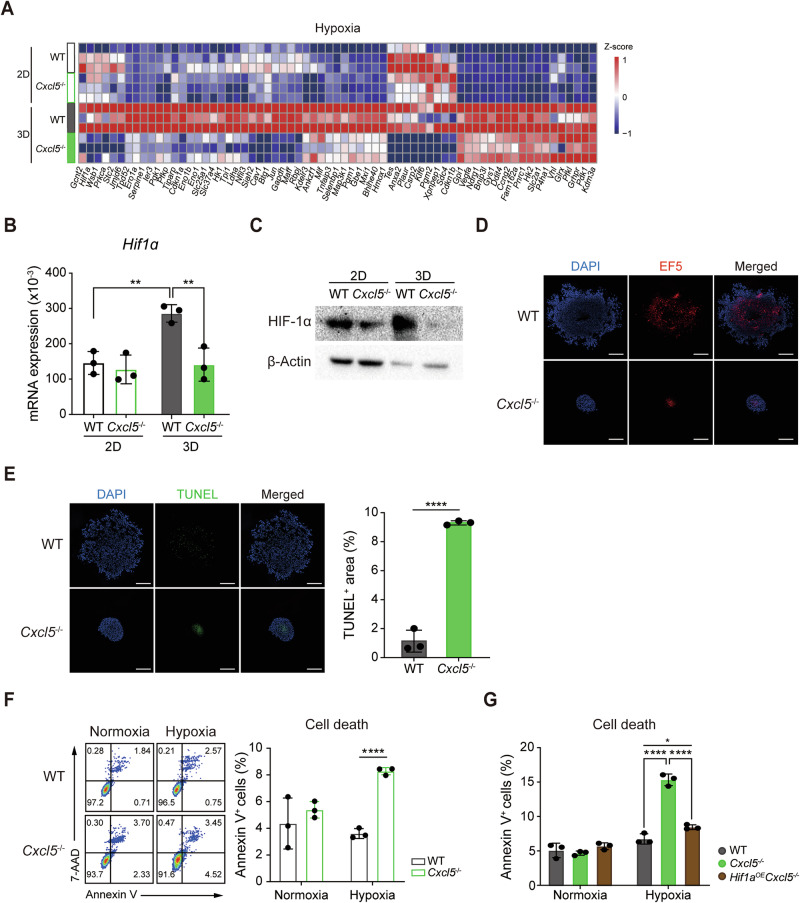
*Cxcl5*^−/−^ cells are susceptible to hypoxia-induced cell death due to defective upregulation of *Hif1α.* **A** Heatmap analysis of hypoxia-related genes. **B**, **C** *Hif1α* expression in 2D- or 3D-cultured WT and *Cxcl5*^−/−^ Panc02 cells assessed by qRT-PCR (**B)** and western blotting (**C)** (mean ± SEM, *n* = 3; 9–12 spheroids per replicate). **D** Immunofluorescence micrographs (left) showing hypoxic regions of WT and *Cxcl5*^−/−^ Panc02 spheroids detected with Cyanine 5-conjugated anti-EF5 antibody (red). Nuclei (blue) were visualized with DAPI. **E** Immunofluorescence micrographs (left) showing cell death as detected by TUNEL staining (green). Bar graph (right) showing the percentage of TUNEL-positive cells (mean ± SEM, *n* = 3). **F** Flow cytometry plot (left) showing annexin V^+^ cells (WT and *Cxcl5*^−/^^−^) cultured in 2D normoxic or hypoxic conditions. Bar graph (right) showing the percentages of annexin V^+^ cells (mean ± SEM, *n* = 3). **G** Bar graph showing the percentage of WT, *Cxcl5*^−/−^, and *Hif1α*^OE^*Cxcl5*^−/−^ cells cultured in 2D normoxic or hypoxic conditions (mean ± SEM, *n* = 3). **B**–**G** Data are representative of three independent experiments. Significance was assessed via two-way (**B)** or one-way ANOVA (**F**, **G)** with Tukey’s post-hoc tests or via unpaired two-tailed Student’s *t*-tests (**E)**. **D,** **E** Magnification, 20X. Scale bar = 200 μm.

Under hypoxic conditions, cells undergo a metabolic shift from oxidative phosphorylation to glycolysis to sustain energy production and biomass synthesis [7]. Our gene expression profiling showed that many genes related to hypoxia were upregulated in 3D-cultured compared to 2D-cultured WT cells. However, 3D-cultured *Cxcl5*^−/−^ cells impaired induction of majority of these genes including alpha subunit of hypoxia inducible factor 1 (*Hif1α*) (Fig. 3A–C). HIF1 is a master transcription factor regulating hypoxic response including the expression of glycolytic enzymes [10, 23, 24]. By using the hypoxia marker EF5, we detected hypoxia inside both WT and *Cxcl5*^−/−^ spheroids (Fig. 3D). Notably, terminal deoxynucleotidyl transferase dUTP nick end labeling (TUNEL)-staining revealed more cell death in the cores of 3D-cultured *Cxcl5*^−/−^ spheroids than WT spheroids (Fig. 3E). These results suggest that although both WT and *Cxcl5*^−/−^ spheroids are subject to hypoxia-induced cell death, *Cxcl5*^−/−^ spheroids are more susceptible to it than WT spheroids. To verify this, we examined cell death in WT and *Cxcl5*^−/−^ cells grown in normoxic or hypoxic 2D culture. We observed that while both WT and *Cxcl5*^−/−^ cells showed similar levels of cell death in normoxia, only *Cxcl5*^−/−^ cells showed increased cell death in hypoxia (Fig. 3F). Moreover, *Hif1α*^*OE*^*Cxcl5*^−/−^ cells that overexpress *Hif1α* stably showed similar resistance to hypoxic cell death as WT cells (Fig. 3G). These results demonstrated that *Cxcl5*^−/−^ cells in the spheroid are more susceptible to hypoxic cell death, suggesting that hypoxia resistance may be the critical factor that distinguishes the growth of 3D-cultured WT and *Cxcl5*^−/^^−^ cells.

### 3D-cultured *Cxcl5*^−/−^ cells exhibit increased mitochondrial ROS and ferroptosis

**Fig. 4:**
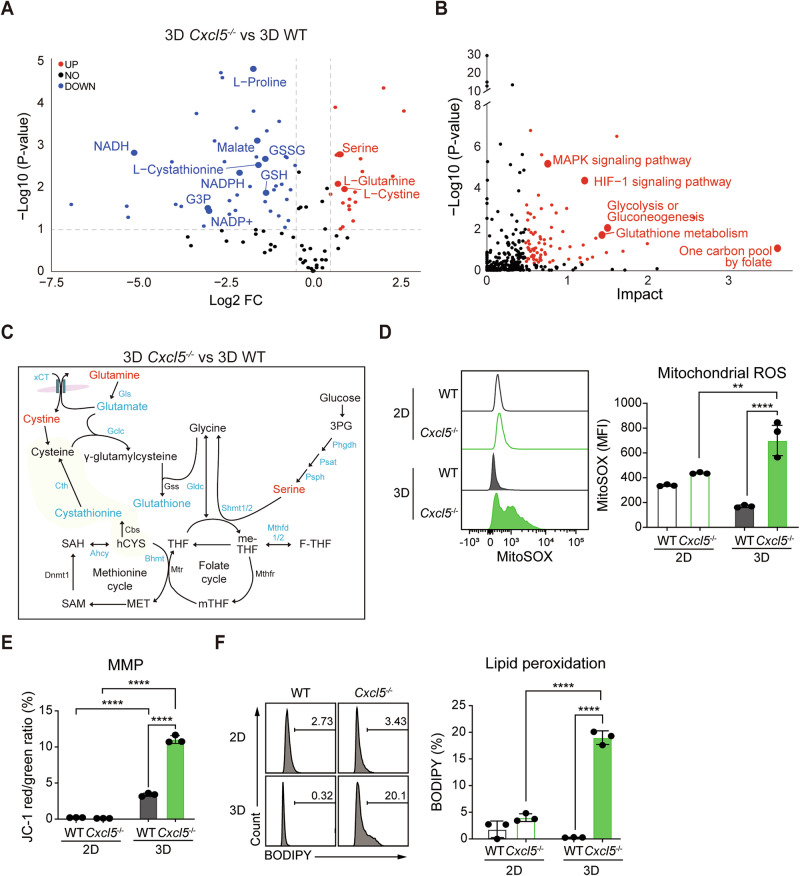
*Cxcl5* deficiency impairs one-carbon metabolism, increasing oxidative stress responses. **A** Volcano plot showing metabolites observed at significantly different levels in the comparison of 3D-cultured *Cxcl5*^−/−^ cells to 3D-cultured WT cells. **B** Joint pathway analysis combining DEGs and differential metabolites using MetaboAnalyst 6.0. The metabolic pathways with *p* < 0.1 and an impact score > 0.5 appear in red. **C** Schematic of one-carbon metabolic pathways showing up- (red) or down-regulated (blue) genes and metabolites in the comparison between 3D-cultured *Cxcl5*^−/−^ cells and 3D-cultured WT cells. The trans-sulfuration process appears in yellow. THF Tetrahydrofolate, me-THF 5,10-methyleneTHF, mTHF 5-methylTHF, F-THF 10-formylTHF, MET Methionine, SAM S-adenosylmethionine, SAH S-adenosylhomocysteine, hCYS homocysteine. **D** Flow cytometry plot showing mitochondrial ROS stained with MitoSOX^TM^ Red (left) and the MFI of MitoSOX^TM^ Red (right, mean ± SEM, *n* = 3). **E** MMP polarization was analyzed by flow cytometry and measured as the ratio of red (aggregates) and green (monomers) JC-1 dye fluorescence (mean ± SEM, *n* = 3). **F** Flow cytometry plots (left) showing lipid peroxidation detected as oxidized BODIPY-C11 signals. The percentages of oxidized BODIPY-C11^+^ cells are also shown (right, mean ± SEM, *n* = 3). For 3D-cultured samples of **D–F**, 12–18 spheroids were pooled per replicate. Data are representative of three independent experiments. Significance was assessed via two-way ANOVA with Tukey’s post-hoc tests.

Next, we took a metabolomics approach to investigate the metabolic change that accompany the alteration in gene expression we observed, looking first at the differences between 2D- and 3D-cultured WT cells. As expected from our transcriptome analysis, we found 3D-cultured WT cells exhibited significant increases in glycolytic intermediates level, such as glyceraldehyde 3-phosphate (GA3P), dihydroxyacetone (DHAP), and glycerol 3-phosphate (G3P). There were also increases in glycerate 3-phosphate (3PG) and phosphoenolpyruvate (PEP), although the increases were less pronounced (Table S3). In contrast, 3D-cultured *Cxcl5*^−/−^ cells showed significantly decreased G3P and decreasing trends for GA3P, 3PG, PEP, and DHAP (Table S3). Importantly, when comparing 3D-cultured *Cxcl5*^−/−^ cells with WT cells, we observed reduced glutathione, L-cystathionine, and NADPH but increased cystine, glutamine, and serine (Fig. 4A). Furthermore, in a joint pathway analysis using MetaboAnalyst 6.0, we found that the genes and metabolites reduced in 3D-cultured *Cxcl5*^−/−^ cells are associated with MAPK signaling, HIF1 signaling, glycolysis, glutathione metabolism, and one carbon pool by folate (Fig. 4B). Indeed, 3D-cultured *Cxcl5*^−/−^ cells showed reduced expression of genes associated with glutathione synthesis (Fig. 4C). For example, glutaminase (*Gls*) and its product glutamate were both reduced. 3D-cultured *Cxcl5*^−/−^ cells also showed reduced levels of the glutamate-cysteine ligase catalytic subunit (*Gclc*) and glutathione. These results indicate that, under 3D culture conditions, CXCL5 upregulates genes that control de novo serine/glycine synthesis, one-carbon metabolism, and glutathione synthesis.

Considering glutathione’s role in redox homeostasis, we hypothesized that the reduced glutathione of 3D-cultured *Cxcl5*^−/−^ cells would be insufficient to properly regulate reactive oxygen species (ROS), leading to increased cell death. When we examined mitochondrial ROS levels using MitoSOX, we found higher levels of mitochondrial ROS in 3D *Cxcl5*^−/−^ cells than WT cells (Fig. 4D). Since mitochondrial membrane potential (MMP) influences mitochondrial ROS production [25], we analyzed MMP using the dye JC-1. 3D-cultured *Cxcl5*^−/−^ cells showed increased JC-1 aggregation indicating MMP hyperpolarization (Fig. 4E). Increased ROS levels and MMP hyperpolarization are associated with ferroptosis, a type of cell death caused by the accumulation of peroxidated lipids [26]. Indeed, we found 3D-cultured *Cxcl5*^−/−^ cells exhibit increased lipid peroxidation (Fig. 4F). Importantly, we found no difference in the activation of caspase-3 and caspase-1, the effector molecules for apoptosis and pyroptosis, respectively, between 3D WT and *Cxcl5*^−/−^ cells (Supplementary Fig. 3A, B). Interestingly, we found that 3D tumor cells exhibited the oligomerization of dihydrolipoamide S-acetyltransferase (DLAT), a characteristic of cuproptosis [27] whereas 2D tumor cells did not. Nevertheless, 3D WT and *Cxcl5*^−/−^ cells did not differ in their levels of DLAT oligomer (Supplementary Fig. 3C). Altogether, our findings indicate that although 3D condition triggers various cell death pathways including apoptosis and cuproptosis, they are not regulated by CXCL5. Rather, our results demonstrate that the primary cause of cell death in 3D *Cxcl5*^−/−^ cells is the ferroptosis-dependent cell death pathway. Furthermore, these results suggest the link between the attenuated one-carbon metabolism of 3D-cultured *Cxcl5*^−/−^ cells and their increased susceptibility to ferroptotic cell death.

### *Hif1α* or *Myc* overexpression restricted ferroptosis and restored growth of 3D-cultured *Cxcl5*^−/−^ cells

**Fig. 5:**
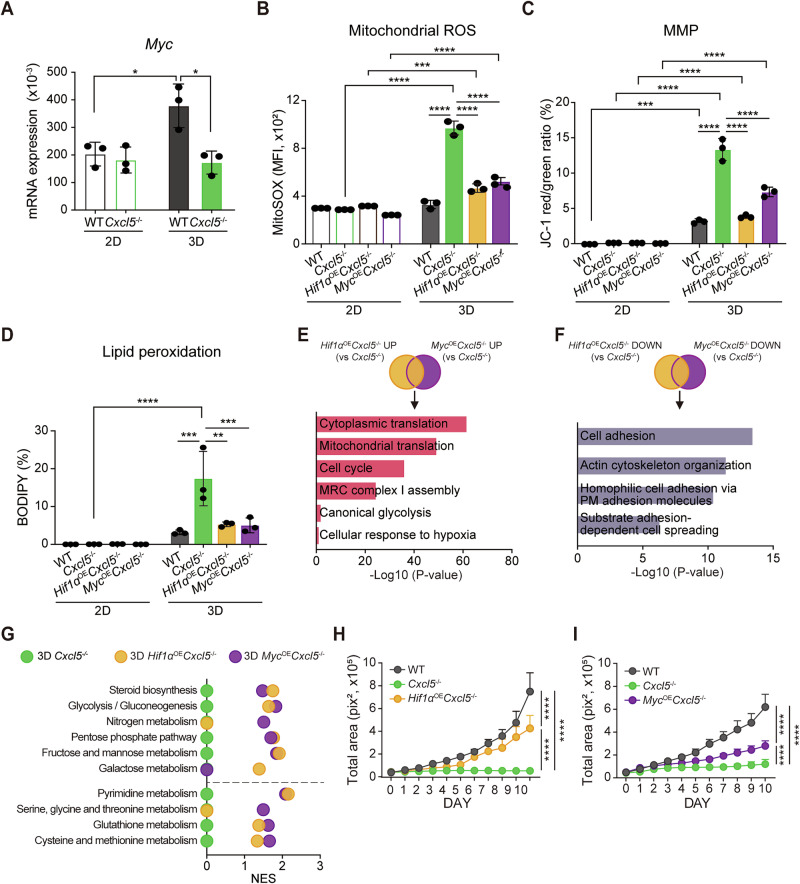
Overexpression of *Hif1α* and *Myc* successfully control the oxidative stress and reverse impaired cancer metabolism and tumor spheroid growth. **A** *Myc* mRNA levels in 2D- or 3D-cultured WT and *Cxcl5*^−/−^ cells assessed by qRT-PCR (mean ± SEM, *n* = 3). **B** Mitochondrial ROS levels detected with MitoSOX^TM^ Red. **C** MMP polarization was measured as the ratio of red/green JC-1 dye fluorescence. **D** Lipid peroxidation percentages were measured via oxidized BODIPY-C11-positive cells in 2D- or 3D-cultured WT, *Cxcl5*^−/−^, *Hif1α*^*OE*^*Cxcl5*^−/−^, and *Myc*^*OE*^*Cxcl5*^−/−^ cells (mean ± SEM, *n* = 3). **E**, **F** GO analysis of commonly upregulated (**E)** or downregulated (**F)** DEGs in *Hif1α*^OE^*Cxcl5*^−/−^ cells and *Myc*^OE^*Cxcl5*^−/−^ cells compared to *Cxcl5*^−/−^ cells. **G** GSEA of metabolic pathways showing differences between 3D-cultured *Cxcl5*^−/−^ (green), *Hifα*^*OE*^*Cxcl5*^−/−^ (yellow) and *Myc*^*OE*^*Cxcl5*^−/−^ cells (purple). **H, I** Total areas of WT, *Cxcl5*^−/−^, and *Hifα*^*OE*^*Cxcl5*^−/−^ (**H)** or *Myc*^*OE*^*Cxcl5*^−/−^ cell spheroids (**I)** (*n* = 9 spheroids/group). For 3D-culture samples of **A,** 9–15 spheroids were pooled per replicate while 12–18 spheroids were pooled per replicate for **B–D**. Data are representative of three independent experiments (mean ± SEM). Significance was assessed via two-way ANOVA with Tukey’s post-hoc tests.

Serine/glycine biosynthesis and one-carbon metabolism are regulated by multiple transcription factors, including HIF-1A, MYC, NFE2L2 (aka NRF2), and ATF4 [28]. We observed 3D-cultured WT cells express higher levels of *Myc* than 2D-cultured WT cells, but loss of *Cxcl5* blocks this upregulation of *Myc* at the transcript (Fig. 5A) and protein (Supplementary Fig. 4A) levels. We therefore tested whether overexpression of *Hif1α* or *Myc* (*Hif1α*^*OE*^*Cxcl5*^−/−^ and *Myc*^*OE*^*Cxcl5*^−/−^ cells) can reverse the oxidative damage in 3D-cultured *Cxcl5*^−/−^ cells. Interestingly, we found that both *Hif1α*^*OE*^*Cxcl5*^−/−^ and *Myc*^*OE*^*Cxcl5*^−/−^ cells showed a rescue of the redox imbalance, hyper-MMP, and increased lipid peroxidation we observed in 3D-cultured *Cxcl5*^−/−^ cells (Fig. 5B–D). These results suggest HIF-1α and MYC play a critical role in maintaining redox homeostasis and mitochondrial function in 3D culture.

Since overexpression of *Hif1α* or *Myc* conferred oxidative stress resistance on *Cxcl5*^−/−^ cells, we examined the differences between their effects on metabolism and 3D growth. RNA sequencing and GO analysis of WT, *Cxcl5*^−/−^, *Hif1α*^*OE*^*Cxcl5*^−/−^, and *Myc*^*OE*^*Cxcl5*^−/−^ cells revealed the biological process including cell cycle (*Cdk1, Ccnb1, Cdc6*), glycolysis (*Hk2, Aldoa, Pgk1, Ldha*), and cellular responses to hypoxia (*Ero1α, Hif1α, Eif4ebp1*) were commonly upregulated by overexpression of *Hif1α* and *Myc* (Fig. 5E, Supplementary Fig. 4B). Glycolytic metabolites downregulated in 3D-cultured *Cxcl5*^−/−^ cells, including G3P, 1,3-bisphosphoglycerate (1,3BPG), 3PG, and PEP, were also restored by *Hif1α* or *Myc* overexpression (Supplementary Fig. 4B). In contrast, overexpression of *Hif1α* or *Myc* reduced the genes involved in cell adhesion and actin cytoskeleton organization which were up-regulated in 3D-cultured *Cxcl5*^−/−^ cells (Fig. 5F). In a GSEA analysis, we found that *Hif1α* or *Myc* overexpression restored most of the metabolic pathways downregulated by *Cxcl5* deletion (Fig. 5G). Interestingly, only *Myc* but not *Hif1α* overexpression restored the expression of the genes for serine, glycine, and threonine metabolism and the genes for nitrogen metabolism. Meanwhile, only *Hif1α* but not *Myc* overexpression rescued the expression of the genes for galactose metabolism. Further supporting our findings from the transcriptomic analysis, our metabolomics analysis shows that *Hif1α* or *Myc* overexpression recovered the metabolites in the metabolic pathways whose gene expressions are demonstrated to be downregulated by *Cxcl5* deficiency in our RNA-seq data (Supplementary Fig. 4C, D). In addition, *Hif1α* or *Myc* overexpression in *Cxcl5*^−/−^ cells (*Hif1α*^*OE*^*Cxcl5*^−/−^ or *Myc*^*OE*^*Cxcl5*^−/−^*)* partially rescued the reduced 3D spheroid growth of *Cxcl5*^−/−^ cells (Fig. 5H, I, Supplementary Fig. 4E, F).

Together, these results demonstrate that *Hif1α* or *Myc* could effectively rescue the many metabolic pathways attenuated by *Cxcl5* deficiency. Specifically, *Myc* overexpression induced a stronger rescue of serine/glycine biosynthesis and one-carbon metabolism than *Hif1α*. These rescued pathways may confer a growth advantage on tumor spheroids by providing both energy and building blocks and by protecting tumor cells from oxidative cell death.

### PAAD patients expressing higher levels of CXCL5-correlated genes associated with hypoxia and metabolism have poorer prognosis

**Fig. 6:**
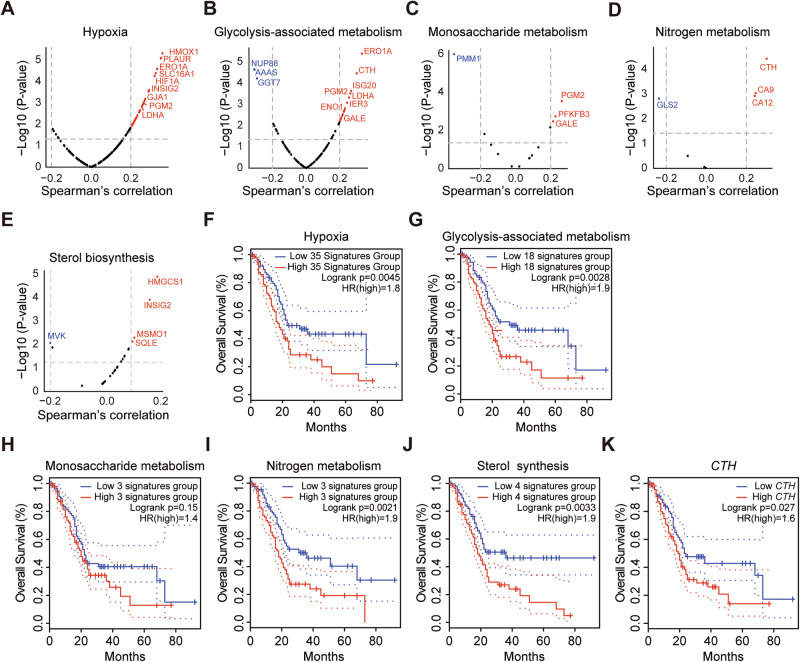
The clinical impact of CXCL5-correlated genes. **A**–**E** CXCL5 expression is correlated with many genes associated with hypoxia (**A)**, glycolysis-related pathways (e.g., glycolysis, PPP, serine/glycine biosynthesis, one-carbon metabolism, and glutathione metabolism) (**B)**, monosaccharide metabolism (**C)**, nitrogen metabolism (**D)**, and sterol biosynthesis (**E)**. **F**–**K** The overall survival of PAAD patients with different expression levels of the genes mentioned in **A**–**E**. The corresponding metabolic pathways are above the plots.

Next, we investigated the impact of *Cxcl5*-dependent genes on the survival of pancreatic adenocarcinoma (PAAD) patients. From the PAAD dataset available in cBioPortal, we identified 19,848 genes whose expression is correlated with CXCL5 levels. We then narrowed this list to 2490 genes homologous to mouse genes that are up-regulated in 3D-cultured WT but not *Cxcl5*^−/−^ cells and categorized them in metabolic pathway gene set collections using the Molecular Signatures Database (MSigDB). A positive correlation between the gene set collections and CXCL5 expression was evaluated based on Spearman’s correlation (Fig. 6A–E, Table S4). We found that PAAD patients with higher expression of these gene set collections fared worse overall survival (Fig. 6F–J). We noted that cystathionine gamma-lyase (*CTH*), which produces cysteine by hydrolyzing cystathionine [29], is commonly associated with glycolysis, serine/glycine biosynthesis, one-carbon metabolism, and nitrogen metabolism. Higher expression of *CTH* correlated with poor PAAD patient survival (Fig. 6K). Considering the correlation between CXCL5 and CTH expression and its association with the metabolism regulation and cancer progression, CXCL5-CTH axis may orchestrates the overall metabolic programming and 3D cancer progression, which warrants further investigation. Together, these results prove that *Cxcl5* expression is correlated with genes associated with hypoxia and a broad range of metabolic pathways that seem to affect PAAD patient survival.

### *HIF1A* or *MYC* overexpression may restrict the efficacy of drug targeting CXCR2

**Fig. 7:**
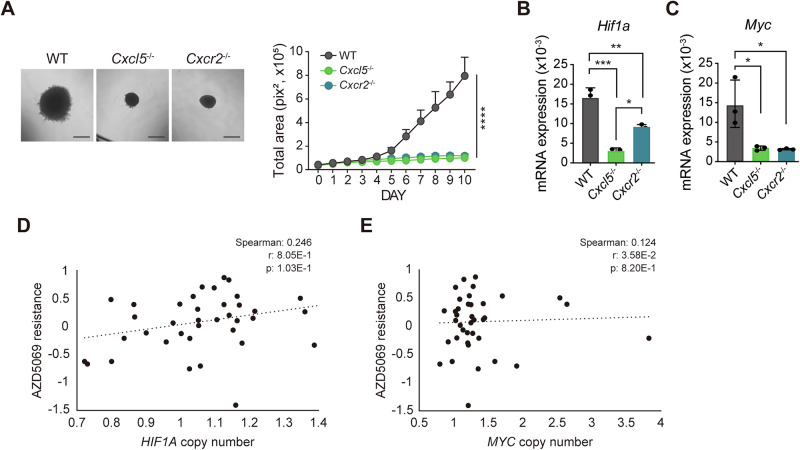
*Hif1α* and *Myc* may inhibit the efficacy of drug targeting CXCR2. **A** Bright field micrographs (left) of WT, *Cxcl5*^−/−^, and *Cxcr2*^−/−^ spheroids. Magnification, 4X. Scale bar = 500 μm. Bar graph (right) showing total spheroid area (*n* = 6 spheroids/group). **B**, **C** *Hif1α* (**B)** or *Myc* (**C)** mRNA levels in 3D-cultured WT, *Cxcl5*^−/−^, and *Cxcr2*^−/−^ cells assessed by qRT-PCR (*n* = 9–12 spheroids/group). **D,** **E** Data from the DepMap portal [34] show the resistance of various human PDAC cancer cell lines to AZD5069 increasing with HIF1A (**D)** or MYC (**E)** copy number. Drug resistance is indicated as a log2-fold-change in the Profiling Relative Inhibition Simultaneously in Mixtures (PRISM) score from repurposed public 23Q2 data [57]. Log2-transformed gene copy numbers (relative to ploidy +1) were extracted from the DepMap database (Copy Number Public 23Q4) [58]. Each dot represents an individual PDAC cell line. Dotted lines represent a linear regression of gene copy number versus drug resistance. *r* regression, *p* *p*-value. **A**–**C** Data are representative of three independent experiments and presented as mean ± SEM. Significance was assessed via two-way (**A)** and one-way (**B,** **C)** ANOVAs with Tukey’s post-hoc tests.

CXCL5 binds the chemokine receptor CXCR2 and facilitates tumor proliferation, invasion, and angiogenesis via various signaling pathways in different types of cancer [30]. Indeed, *CXCL5* expression levels are higher in late-stage PDAC (Supplementary Fig. 5A), correlating with poor overall patient survival (Supplementary Fig. 5B). Moreover, CXCR2 is implicated in PDAC progression [31, 32]. We therefore expected that CXCL5 regulates *Hif1α* and *Myc* expression through CXCR2. Indeed, loss of *Cxcr2* recapitulated the growth inhibition we observed in *Cxcl5*^−/−^ spheroids (Fig. 7A). 3D culture of *Cxcr2*^−/−^ cells also failed to induce *Hif1α* and *Myc* expression (Fig. 7B, C). Since drugs targeting CXCR2 have failed to prove effective in the clinic [33], we reasoned cancer cells expressing high levels of *HIF1A* or *MYC* are resistant to CXCR2 inhibitors. Using the DepMap portal [34], we found increased copy number of *HIF1A* or *MYC* is correlated with a reduced sensitivity of various human PDAC cell lines to the CXCR2 targeting drug AZD5069 (Fig. 7D, E). This supports the possibility that cancers can develop resistance to CXCR2 inhibition via the upregulation of *HIF1A* or *MYC* expression and suggest that combining drugs targeting CXCR2 and *HIF1A* or *MYC* may increase their therapeutic efficacy.

## Discussion

In this study, we established a tumor spheroid-in-Matrigel culture system to model 3D tumor growth, which, in the TME, is regulated by physical and chemical interaction with the ECM. We identified a significant upregulation of *Cxcl5* expression in M1-CM-treated spheroids. While CXCL5 secreted by immune cells or fibroblasts reportedly plays an important role in modulating the composition and activities of immune cells in the TME [30, 31], little is known about the role cancer-derived CXCL5 plays in cancer progression. Here, we show that *Cxcl5* plays the role of a conductor orchestrating the global metabolic remodeling necessary for cancer growth and survival in the 3D TME. More specifically, *Cxcl5*-deficient cancer cells exhibit reduced expression of enzymes and metabolites pertaining to glycolysis, serine/glycine biosynthesis, and one-carbon metabolism. This reduction is due to the impaired upregulation of *Hif1α* and *Myc*, which are master transcription factors that regulate the metabolic pathways mentioned above. These glycolysis-associated pathways provide building blocks required for cell growth and division, and anti-oxidants that protect against ROS-mediated cell death.

One-carbon metabolism not only generates glutathione but also contributes to the production of nucleic acids and methyl groups used in post-translational modifications through the folate and methionine cycles [28]. Metabolic intermediates from glycolysis are used to generate NADPH for redox homeostasis through PPP and the folate cycle [7, 10]. Therefore, we suspect the spheroid growth defect of *Cxcl5*-deficient cancer cells may result from a combined deficit in energy, biomass, and antioxidant production. We found that the reduced levels of antioxidant metabolites in 3D *Cxcl5*^−/−^ cells make them more susceptible to ferroptosis secondary to inadequate quenching of mitochondrial ROS. Recently, ferroptosis was spotlighted as a promising therapeutic target in the treatment of cancers resistant to conventional pro-apoptotic treatments [35]. We showed that *Cxcl5* deletion significantly increased ferroptosis in 3D conditions, suggesting CXCL5 signaling as a potential target for the induction of tumor ferroptosis.

While translational and post-translational mechanisms of HIF-1α and MYC regulation are well-described [36, 37], the transcriptional mechanisms of *Hif1α* and *Myc* regulation are less clear. *Hif1α* is transcriptionally regulated by transcription factors SP1, STAT3, NF-κB [38, 39], protein arginine methyltransferase 1 (PRMT1) [40], and by non-coding RNAs [41]. *Myc* transcription and mRNA levels are controlled by transcription factors, including CCHC-type zinc finger nucleic acid binding protein (CNBP), far upstream element (FUSE) binding protein (FBP), and T cell factor (TCF), as well as by miRNAs [42]. The *Myc* promoter is activated through the resolution of torsional stress caused by promoter supercoiling. Stress at the FUSE is relieved with the binding of FBP, while stress at the nuclease hypersensitive element III_1_ (NHE III_1_), which contains a DNA guanine quadruplex (G4) structure, is relieved with the binding of SP1, CNBP, and hnRNP K [43]. Given that the *Hif1α* promoter also contains a G4 structure [44], it will be interesting to examine whether CXCL5-CXCR2 signaling leads to the activation of G4-resolvase complexes.

In addition, we showed *Hif1α* overexpressing cancer cells were able to induce galactose metabolism while *Myc* overexpressing cancer cells did not. However, *Myc* overexpressing cancer cells had superior effect on positively regulating multiple metabolic pathways including glycine, serine, threonine, glutamine, and nitrogen metabolism over *Hif1α* overexpression. Many studies have indicated that the cancer metabolic pathways communicate with cell death processes [45–47]. Although we identified significant impairment of various metabolic pathways with *Hif1α* and *Myc* downregulation in 3D *Cxcl5*-deficient cells compared to WT cells, only ferroptosis significantly increased in the 3D *Cxcl5*-deficient cells. Our results indicate that CXCL5-dependent metabolic regulation contributes to the suppression of ferroptosis rather than other cell death pathways including apoptosis, pyroptosis, and cuproptosis.

Pancreatic cancers are heterogeneous with regard to glycolysis, lipogenesis, and redox pathway activity. This allows PDAC patients to be stratified into several subgroups (i.e., quiescent, glycolytic, cholesterogenic, and mixed) [48, 49]. Understanding a specific pancreatic cancer’s metabolic phenotype is particularly important because differences in metabolic phenotype lead to differences in a cancer’s responsiveness to various types of chemo- and immunotherapies [48, 50]. Our study provided a list of genes associated with hypoxic response and metabolic regulation whose expression showed a correlation with CXCL5 expression. Importantly, we found that PAAD patients expressing higher levels of those CXCL5-correlated genes show poorer prognosis. Thus, these genes could serve as useful biomarkers for selecting the best treatment options and monitoring therapeutic outcomes.

## Materials and methods

### 3D spheroid culture

For spheroid formation, Panc02 mouse pancreatic cancer cells [51] were seeded onto 96-well U-bottom ultra-low attachment plates (Corning). 1000 cells per spheroid were seeded with complete DMEM media (50 μl Dulbecco’s modified eagle media (DMEM) (Welgene) supplemented with 10 mM N-2-hydroxyethylpiperazine-N’-2-ethanesulfonic acid (HEPES), 100 U/ml penicillin/streptomycin, 2 mM L-glutamine, and 10% fetal bovine serum (FBS) (Gibco)). The cells were incubated at 37 °C and 5% CO_2_ for 24 h. Next, 2 mm-thick polydimethylsiloxane (PDMS) sheets with 3 holes were prepared and placed into the wells of a 24-well plate to support the structural stability of our 3D culture system and facilitate spheroid harvest. Then, 20 μl of Matrigel (Corning) was pipetted into the holes and allowed to solidify during 30 min incubation at 37 °C and 5% CO_2_. A tumor spheroid resuspended in 0.5 μl of Matrigel diluted 50:50 with Dulbecco’s phosphate buffered saline (DPBS) was loaded on top of the solidified layer of Matrigel and incubated for 5 min at 37 °C. Then, 15 μl of additional Matrigel was added above the spheroid and incubated for an additional 30 min at 37 °C. After the top layer of Matrigel solidified, 600 μl complete DMEM with or without macrophage conditioned media was added to each well and 6.5% FBS containing DMEM was used for control. The culture media was replaced every three days. For supplementing the CXCL5, recombinant CXCL5 (BioLegend, Cat# 573304) was used at various concentrations indicated in the figures. For experiments on 3D-cultured WT, *Cxcl5*^−/−^, *Hif1α*^OE^, and *Myc*^OE^, 600 μl complete DMEM was applied. Images were captured every day for spheroid growth analysis using an Olympus CKX53 inverted microscope equipped with an eXcope XCAM1080 camera. Images were analyzed using ImageJ (NIH) and ImageJ Fiji (NIH).

### Spheroid dissociation

To dissolve the Matrigel, cell recovery solution (Corning) was added and incubated for 30 min at 4 °C on a rotator. The freed spheroids were then transferred into new tubes and carefully washed with cold DPBS three times to remove the remaining Matrigel. Then, the spheroids were dissociated for 20 min at 37 °C in 200 μl accutase cell detachment solution (Millipore) and then an equal volume of 0.25% pre-warmed trypsin-ethylenediaminetetraacetic acid (EDTA) (Gibco) was added for additional 5 min incubation at 37 °C. The spheroids were pipetted vigorously to achieve complete dissociation. The resulting cells were then washed with ice-cold complete DMEM and used for further studies.

### Preparation of macrophage conditioned media

Bone marrow cells were isolated from female 7-week-old C57BL/6 J mice and cultured for 7 days in macrophage culture media (complete DMEM mixed 7:3 with filtered L-929 cell culture supernatant) to encourage differentiation into macrophages. To confirm macrophage differentiation by flow cytometry, the cells were resuspended in anti-CD16/32 antibody (clone 2.4G2) to block FcγR-mediated non-specific binding and stained with antibodies against CD11b (Cytek Bioscience Cat# 25-0112) and F4/80 (BioLegend Cat# 123116). Data were acquired on an LSR Fortessa (BD Biosciences) and analyzed with FlowJo (BD Biosciences). The sorted cells were collected and re-seeded in 6-well tissue culture plates at 8 ×10^5^ per well in 1.5 ml macrophage culture media. The cells were then incubated overnight at 37 °C and 5% CO_2_ to allow attachment to the bottom of the culture plates. For M1 macrophage differentiation, the cells were incubated in 10 ng/ml IFNγ (PeproTech) for 24 h followed by a second 24 h incubation with 20 ng/ml lipopolysaccharide (LPS) (Sigma). M1 macrophage differentiation was verified by flow cytometry using an anti-CD86 antibody (BioLegend Cat# 105006) and by quantitative real-time polymerase chain reaction (qRT-PCR) with primers specific for inducible nitric oxide synthase (iNOS or *Nos2*). For detecting M2 macrophage differentiation, anit-CD206 antibody was used (BioLegend Cat# 141720). 20 ng/ml TNFα, 20 ng/ml IL-4, or 40 ng/ml TGF-β1 was used to stimulate macrophages. To obtain tumor conditioned media (TCM), the culture supernatants of 2D- or 3D-cultured tumor cells were removed one day before harvest and replaced with serum free media. After 24 h, the serum free media from the 2D (2D TCM) or 3D (3D TCM) tumor cells was harvested. M0-CM was acquired from the culture of unstimulated macrophages. BMDMs were washed with DPBS (Welgene) three times before adding serum free DMEM containing 30% L929 cell supernatant. After 24 h, the culture supernatants were collected and filtered through 0.22 μm polyvinylidene difluoride (PVDF) filters (Merck). Complete DMEM mixed at 1:1 with macrophage conditioned media (CM) was prepared. DMEM containing 6.5% FBS, matching the percentage of FBS in M0- or M1-CM was used as control media.

### Generation of cell lines

Oligonucleotides for single guide RNAs (sgRNAs) targeting mouse *Cxcl5* and human *CXCL5* genes were commercially synthesized (Bioneer) with guide sequences of 5‘-CATCCGCATGAATGGCGAGATGG-3‘ and 5‘-CGGTCGCGGGTTCCTGAACT-3‘, respectively, and were cloned into the lentiCRISPRv2 vector (Addgene, plasmid #52961). To complement *Cxcl5* in *Cxcl5*-deficient cell line, *Cxcl5*-encoding DNA was designed to incorporate a silent mutation on the sgRNA-targeting site, synthesized by Bioneer, and subcloned into a pLPCX retroviral vector (NotI and AgeI). For *Cxcr2* knockout, a *Cxcr2* sgRNA CRISPR/Cas9 all-in-one lentivector was purchased from Applied Biological Materials Inc. (Abm, Cat# 171811140595). To stably overexpress *Hif1α*, a plasmid encoding a stabilized mouse mutant of *Hif1α* (P402A/P577A/N813A) was purchased from Addgene (Addgene, plasmid #44028). Then, *Hif1α*-encoding DNA was amplified by PCR and subcloned into a pLPCX retroviral vector (XhoI and EcoRI). For *Myc* overexpression, a PIG-MYC expressing vector was purchased from Addgene (Addgene, plasmid #177650) and subcloned into a pLPCX vector (XhoI and EcoRI). The plasmids were transfected to HEK293T cells, and after 48 h, the virus-containing supernatants were collected, filtered, and used to infect Panc02 cells (WT for knockout; *Cxcl5*^−/−^ for overexpression). The cells were then selected with puromycin at a concentration of 0.5 μg/ml. *Cxcl5* and *Cxcr2* knockouts were confirmed via immunoblot with anti-CXCL5 (Abcam, ab18134) and flow cytometry with anti-CXCR2 (BioLegend, Cat# 149316) antibodies, respectively. Expression of *Hif1α* and *Myc* was confirmed by qRT-PCR using primers listed in Table S1.

### Cell proliferation assay

Cell numbers in spheroids were determined by fluorometric DNA quantification using the CyQUANT^TM^ cell proliferation assay kit (Invitrogen) according to manufacturer’s instructions. Single spheroids were placed individually in the wells of 96-well plates after washing with DPBS to remove culture media. Alternatively, when analyzing 2D-cultured cell proliferation, the cells were stained with 1 μM CellTrace^TM^ Far Red dye (Invitrogen) according to the manufacturer’s instructions. Far Red dye dilution was measured by flow cytometry on an LSRFortessa^TM^ (BD biosciences) and the resulting data was analyzed using FlowJo. Dilution was evaluated via mean fluorescence intensity (MFI).

### Cell migration assay

Panc02 spheroids treated with macrophage-CM were dissociated and 1 × 10^4^ cells were placed in the 24-well inserts containing 100 μl of serum-free DMEM. On the bottom chamber, 600 μl of complete DMEM was added. After 24 h incubation, the upper side of the insert was wiped with a cotton swab to remove non-migrating cells and cells that reached the bottom of the insert were fixed with 70% ethanol. Then, cells were stained using 1% crystal violet/20% ethanol. The migrating cells were analyzed using microscopy and quantified as cell counts per insert.

### Tumor growth

A total of 1 × 10^5^ WT or 5 ×10^5^ *Cxcl5*^−/−^ Panc02 cells were subcutaneously injected into the left flank of female 7-week-old C57BL/6 J mice in 100 μl of DPBS (Welgene). The mice and their tumors were monitored every three days until the mice were sacrificed on day 10 after tumor injection. Tumor size was measured using a digital caliper (Mitutoyo); tumor volumes were estimated according to the following formula: (length x width^2^)/2.

### Quantitative real-time polymerase chain reaction (qRT-PCR)

Total RNA was extracted using TRI Reagent^TM^ (Thermo) and reverse transcribed with oligo dT to generate complementary DNA using SuperiorScript III reverse transcriptase (Enzynomics). RbTaq^TM^ SYBR qPCR 2X PreMIX (SYBR Green with low ROX) (Enzynomics) was used to amplify target genes and PCR was carried out on a Rotor-Gene^®^ Q real-time PCR cycler (Qiagen). Target gene expression was normalized to that of β-actin. Primers are listed in Table S1.

### RNA-sequencing

RNA-seq libraries were prepared using Illumina TruSeq Stranded total RNA sample preparation kits. The resulting libraries were sequenced on an Illumina NovaSeq with pair-end 101 bp reads by the sequencing service provider (Macrogen). The reads were aligned with the GRCm38 mouse reference genome using HISAT2 [52]. Then, the Stringtie algorithm [53] was used to assemble the aligned reads and calculate read counts in each sample for each transcript. Differentially expressed genes (DEGs) were determined using the Deseq2 package (ver. 1.32.0) in R (ver. 4.1.0) and fed into a Gene Ontology (GO) analysis using the Database for Annotation, Visualization and Integrated Discovery (DAVID) and a pathway enrichment analysis using the Kyoto Encyclopedia of Genes and Genomes (KEGG) databases [54]. Genes associated with hypoxia and metabolism were obtained from MSigDB [55] and the correlation of their expression with *CXCL5* expression in PAAD patients was identified using cBioPortal [56].

### Western blotting

Cells or spheroids were lysed in 100 μl of RIPA lysis buffer containing 25 mM Tris-HCI (pH 7.6), 150 mM NaCl, 1% NP-40, 1% sodium deoxycholate, 0.1% sodium dodecyl sulfate (SDS) (Thermo), and a supplement of protease and phosphatase inhibitors (Roche) and centrifuged for 15 min at 13,000 rpm at 4 °C to remove cell debris. The supernatants, e.g., the cell lysate, were boiled for 5 min at 95 °C in a sample buffer containing 1% SDS, 10% glycerol, and 0.05% bromophenol blue (BPB) in 6.25 mM Tris buffer (pH 6.8) with 5% β-mercaptoethanol. To analyze DLAT oligomerization, the cell lysates were prepared in a sample buffer without β-mercaptoethanol. Cell lysates were separated by 10% SDS-polyacrylamide gel electrophoresis (PAGE) and transferred onto polyvinylidene difluoride (PDVF) membranes. For analyzing CXCL5, nitrocellulose membranes (Millipore) was used. The membranes were incubated in a Tris-buffered saline blocking solution (Tris base, 24.7 mM; sodium chloride, 137 mM; potassium chloride, 2.7 mM) containing 5% nonfat dried milk and 0.1% Tween 20 for 30 min at room temperature (RT). Then, the membranes were incubated for 2 h at RT with the following antibodies diluted in blocking solution: anti-CXCL5 (1:1000), anti-HIF-1α (1:500; Abcam, ab179483), anti-cMYC (1:1000; Cell Signaling Technology, #9402), anti-caspase-3 (1:1000; Cell Signaling Technology, #9662), anti-caspase-1 (1:1000; Cell Signaling Technology, #83383), anti-cleaved caspase-1 (1:1000; Cell Signaling Technology, #89332), anti-DLAT (1:1000; Abcam, ab110333), anti-β-actin (1:1000; Abcam, ab8226), and anti-GAPDH (1:1000, Santa Cruz Biotechnology, sc-166545). Uncropped western blots can be found in Supplementary Fig. 6.

### Immunofluorescence staining

Spheroids were fixed in fixation buffer containing 4% paraformaldehyde (BioLegend) for 2 h at RT. They were then washed with PBS, transferred to 30% sucrose for 3 h at RT, and then embedded in Optimal Cutting Temperature compound (Sakura Finetek) and frozen at -80 °C. The spheroids were cut into 10 μm sections, applied to slides, washed in PBS three times, and blocked in PBS containing 5% goat serum (Vector Laboratories) and 5% bovine calf serum for 30 min. The sections were then incubated with rhodamine-conjugated phalloidin (Invitrogen) to visualize actin filaments or primary antibodies (1:100) for 1 h at RT. To identify hypoxic regions, the spheroids were cultured with the hypoxia marker EF5 [2-(2-nitro-1*H*-imidazole-1-yl)-*N*-(2,2,3,3,3-pentafluoropropyl)-acetamide] (Sigma-Aldrich) for 24 h before spheroid harvest. The EF5 was then detected with an anti-EF5 antibody (Millipore Cat# EF5012-250UG). To identify cell death, an in situ cell death detection kit (Roche) was used according the manufacturer’s instructions. Briefly, the spheroid sections were stained with a 1:9 mixture of enzyme and labeling solutions for 1 h at 37 °C after a 2 min RT permeabilization with 0.1% Triton X-100 in 0.1% sodium citrate. Samples were visualized using a LSM980 confocal microscope (ZEISS) and the resulting images were processed with ImageJ or ImageJ Fiji.

### Mitochondrial ROS and membrane potential (MMP) measurements

MitoSOX^TM^ Red (Invitrogen) was used to detect mitochondrial ROS levels according to the manufacturer’s instructions. Cells were stained with 2.5 nM MitoSOX^TM^ in Opti-MEM (Thermo) at 37 °C for 30 min and analyzed by flow cytometry. Mitochondrial membrane potential (MMP) was assessed by 5 mM JC-1 (Invitrogen) staining according to the manufacturer’s instructions. Briefly, cells were stained with Opti-MEM containing JC-1 dye at 37 °C for 30 min. Then, the fluorescence emissions of monomers (green, ~525 nm) and aggregates (red, ~590 nm) were detected by flow cytometry.

### Lipid peroxidation measurements

Cells were stained with 10 μM BODIPY 581/591 C11 dye (Thermo) in complete DMEM media and incubated at 37 °C for 30 min. The cells were then washed with DPBS and analyzed by flow cytometry.

### LC-MS metabolomics

Metabolites were extracted from 2D- or 3D-cultured WT and *Cxcl5*^−/−^ as well as 3D-cultured *Hif1α*^*OE*^*Cxcl5*^−/−^ and *Myc*^*OE*^*Cxcl5*^−/−^ cells. For the 2D culture samples, a total of 1 ×10^6^ WT and *Cxcl5*^−/−^ Panc02 cells were collected. For the 3D culture samples, WT (12 spheroids), *Cxcl5*^−/−^ (36 spheroids), *Hif1α*^*OE*^*Cxcl5*^−/−^ (36 spheroids), and *Myc*^*OE*^*Cxcl5*^−/−^ (24 spheroids) Panc02 spheroids were collected and pooled. All samples were prepared in triplicate. Cell pellets were resuspended in 600 μl of a 2:1 methanol/chloroform mixture for metabolite extraction and then subjected to three mix-freeze-thaw cycles (vortexed for 30 s, frozen in liquid nitrogen for 60 s, and thawed in an ice bath). After adding 400 μl of a 1:1 chloroform/water mixture, the cell lysates were centrifuged at 15,000 *g* for 20 min at 4 °C. The soluble metabolite-containing upper layers were harvested and dried in a centrifugal vacuum evaporator (Vision). The dried samples were dissolved in 100 μl of a 1:1 (v/v) mixture of high performance liquid chromatography (HPLC)-grade acetonitrile and water and subjected to chromatographic separation on an ACQUITY UPLC BEH Amide Column (1.7 μm, 100 ×2.1 mm; Cat# 186004801, Waters) at 40 °C. This was facilitated by the integration of Acquity ultra performance liquid chromatography (UPLC) (Waters) and then transferred to mass spectrometry using a Q Exactive™ Focus Hybrid Quadrupole-Orbitrap™ Mass Spectrometer (Thermo). The mobile phase was divided in two: phase A, 10 mM ammonium acetate in deionized water adjusted to pH 9.6 with NH_4_OH and phase B, 10 mM ammonium acetate in a 2:8 mixture of deionized water and acetonitrile adjusted to pH 9.6 with NH_4_OH. The elution gradients were precisely programmed as follows: initiation 0% A at 0 min, 0% A at 2 min, 40% A at 7.5 min, 40% A at 16 min, 0% A from 16.5 to 20 min, accompanied by a constant flow rate of 0.2 ml/min. The Q Exactive™ Focus Hybrid Quadrupole-Orbitrap™ MS system was equipped with a Heated Electrospray Ionization (HESI-II) probe and calibrated to the following parameters: sheath gas flow rate of 50 ml/min, auxiliary gas flow rate of 15 ml/min, both gases heated to 350 °C, sweep gas flow rate of 2 ml/min, spray voltage of 2.5 kV, capillary temperature at 320 °C, and S-lens RF level of 50. Metabolites were detected in both negative and positive polarity.

### Statistical analysis

Experiments were conducted on at least three biological replicates in 3 independent experiments. Statistical analyses were performed using GraphPad Prism. Statistical significance was assessed with Student’s *t*-tests or analyses of variance (ANOVAs) with Tukey’s post hoc tests for pairwise comparisons. Statistical significance is indicated with *p*-values ≤ 0.05 (*), ≤ 0.01 (**), ≤ 0.001 (***), ≤ 0.0001 (****); only the samples showing significant difference were indicated.

## Supplementary information

Supporting information

Table S1

Table S2

Table S3

Table S4

## Data Availability

The RNA-seq data presented in this study are publicly available in the Gene Expression Omnibus (GEO) at GSE269318. All raw data generated in this study are available upon request.
