# Supplementary material for: Cancer-intrinsic Cxcl5 orchestrates a global metabolic reprogramming for resistance to oxidative cell death in 3D
Source: Cell Death Differ. 2025 Mar 7;32(7):1200–13. doi: 10.1038/s41418-025-01466-y (PMC12284152; doi:10.1038/s41418-025-01466-y)
Supplement: Supplementary file 1 — Supporting information [file 41418_2025_1466_MOESM1_ESM.pdf]

Supplementary Fig 1.

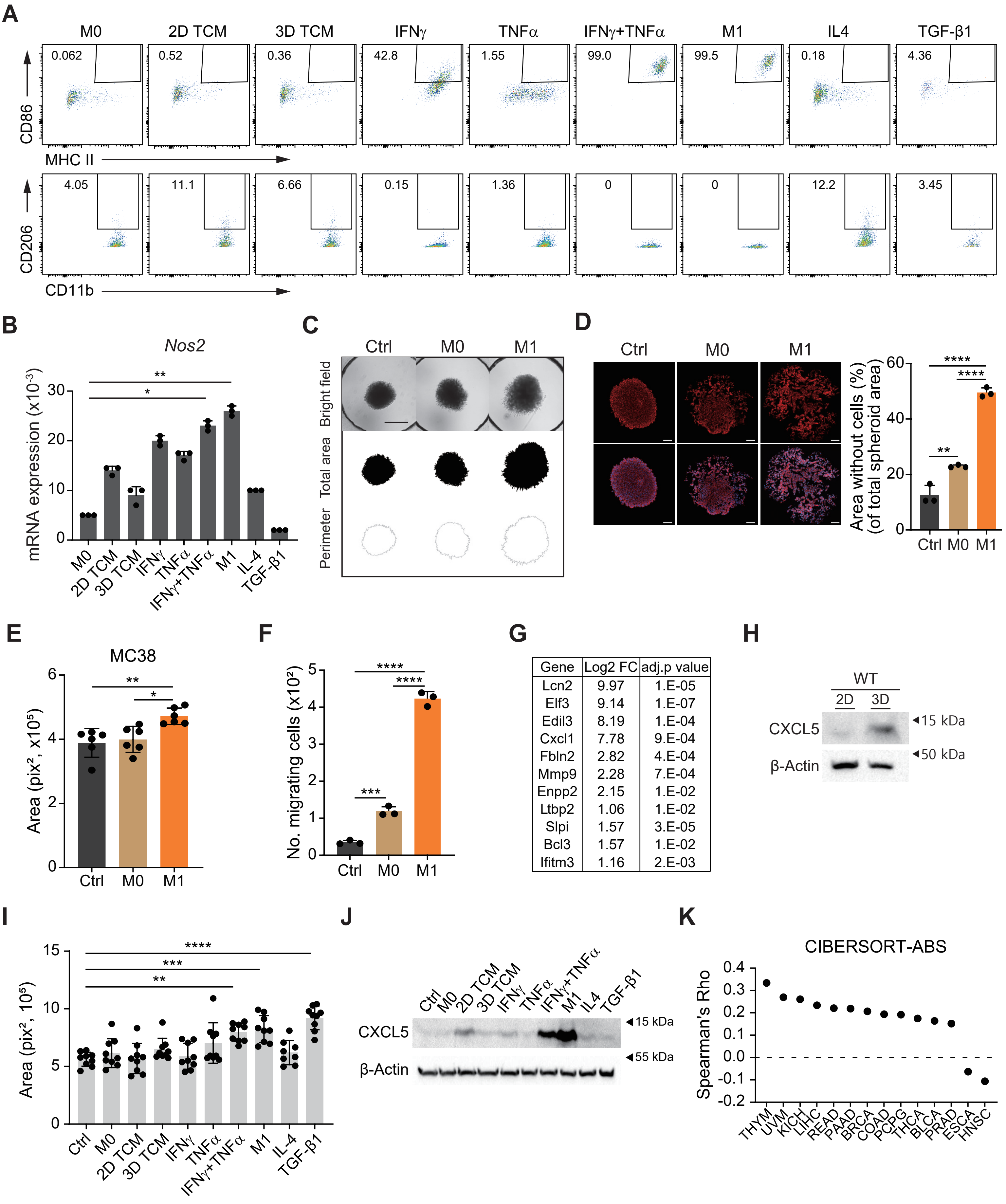

Supplementary Fig 1. IFNγ primed-M1 macrophages promote CXCL5 expression and 3D growth of cancer cells.

A, The polarization of macrophages ($CD11b^+F4/80^+$) by different stimuli is revealed by flow cytometry analysis as either M1 ($CD86^+MHCII^+$) or M2 ($CD206^+$) (n = 3). **B**, *Nos2* mRNA expression in macrophages stimulated as A, assessed by qRT-PCR (n = 3). **C**, Bright field micrographs showing spheroid images (top) analyzed for total area (2nd row, mask image), perimeter (3rd row). Magnification, 4X. Scale bar = 1 mm. **D**, Immunofluorescence micrographs (left) of spheroids stained with phalloidin (red, upper) and 4', 6-diamidino-2-phenylindole (DAPI, blue). Merged images appear below. Magnification, 20X. Scale bar = 100 μm. Bar graph showing empty area percentages (right) (n = 3). **E**, Bar graph showing increased size of MC38 spheroids by M1-CM treatment (n = 6 spheroids/groups). **F**, Bar graph showing enhanced cell migration by M1-CM treatment (n = 3). Cell migration was detected using transwell assay. **G**, Expression of EMT-associated genes that are upregulated 3D tumor cells treated with M1-CM compared to untreated cells. Log2 fold change (Log2FC) and adjusted p values (adj. p value) are shown. **H**, Western blot analysis of CXCL5 expression in 2D- and 3D- cultured Panc02 cells. β-Actin was used for loading control (n = 3). **I**, The conditioned media of macrophages co-stimulated with IFNγ and TNFα promoted tumor spheroid growth (n = 9 spheroids/group). **J**, CXCL5 expression in 3D tumor spheroids grown in the conditioned media of macrophages treated with various stimuli (n = 3). **K**, Correlation between CXCL5 expression and M1 macrophage infiltration in various cancers is shown using TIMER 2.0 based on CIBERSORT-ABS database ($p<0.05$). Data are representative of two or three independent experiments (mean ± SEM). Significance was assessed via non-parametric Kruskal-Wallis ANOVA with Dunn's test (**B**) or one-way ANOVAs with Tukey's post-hoc tests (**D**, **E**, **G**, **I**).

Supplementary Fig 2.

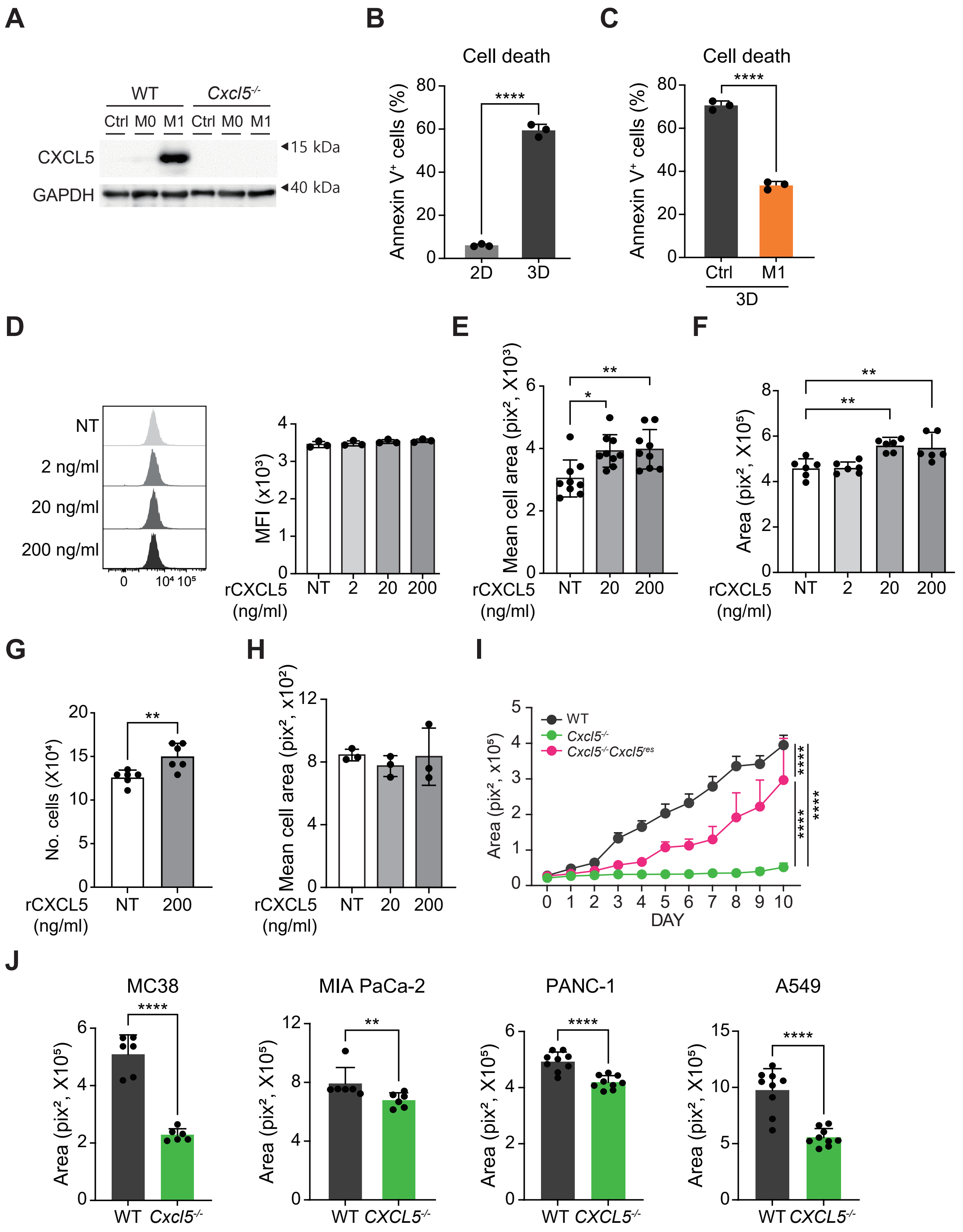

Supplementary Fig 2. CXCL5 complementation restores the growth retardation of *Cxcl5$^{-/-}$* spheroids.

A, Western blot analysis of CXCL5 expression in control (Ctrl)-, M0-CM-, and M1-CM-treated 3D WT and *Cxcl5$^{-/-}$* Panc02 cells. GAPDH was used for loading control (n = 3). **B**, **C**, Bar graph showing the percentages of annexin V^{+} cells cultured in 2D or 3D condition (**B**), Ctrl or M1-CM-treated 3D-cultured Panc02 cells (**C**) (mean ± SEM, n = 3). **D**, Flow cytometry plots (left) showing the proliferation of Panc02 cells grown in 2D with recombinant CXCL5 (rCXCL5), as assessed by Far Red CellTrace^{TM} dye dilutions. Bar graph (right) showing MFI (n = 3). **E**, Bar graph showing mean cell area of individual Panc02 cells grown in 2D with rCXCL5. The mean cell area was calculated by dividing total area of the cells by the cell counts (n = 9). **F**, Bar graph showing the size of Panc02 spheroids treated with rCXCL5 (n = 6 spheroids/group). **G**, rCXCL5 treatment increased cell numbers (per spheroid) of Panc02 spheroids (n = 6 spheroids/group). **H**, Bar graph showing mean cell area of individual cells in the Panc02 spheroids grown with rCXCL5. **(D-H)**, NT, untreated. rCXCL5 concentrations are indicated. MFI, mean fluorescence intensity. **I**, Graph showing total areas of 3D WT, 3D *Cxcl5$^{-/-}$,* and 3D *Cxcl5$^{-/-}$Cxcl5^{res}* *(Cxcl5*-rescued *Cxcl5$^{-/-}$*) Panc02 spheroids (n = 9 spheroids/group). **J**, Various wild-type and CXCL5-deficient mouse and human cancer cell lines are compared in a spheroid area (n = 6-9 spheroids/groups). Significance was assessed via unpaired two-tailed Student's *t*-tests. Data are representative of two independent experiments (mean ± SEM). Significance was assessed via unpaired two-tailed Student's *t*-tests (**B**, **C**, **G**, **J**), one-way ANOVA (**D**, **E**, **F**, **H**), and two-way ANOVA (**I**) with Tukey's post-hoc tests.

Supplementary Fig 3.

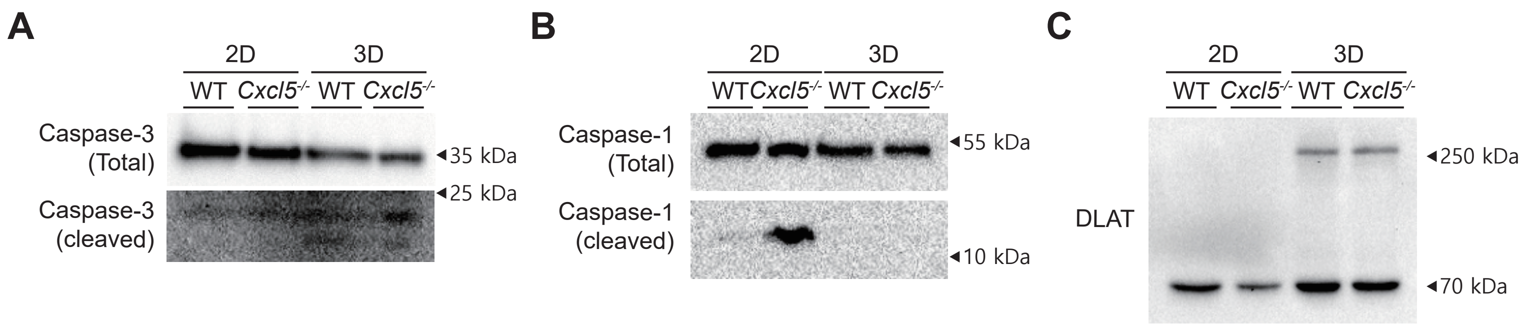

Supplementary Fig 3. No difference in apoptosis, pyroptosis, and cuproptosis between 3D-cultured wild-type and *Cxcl5*-deficient cancer cells.

A, **B**, Western blot analysis of caspase-3 **A**) or caspase-1 (**B**) activation (n = 3). **C**, Western blot analysis of DLAT oligomers (n = 3).

Supplementary Fig 4.

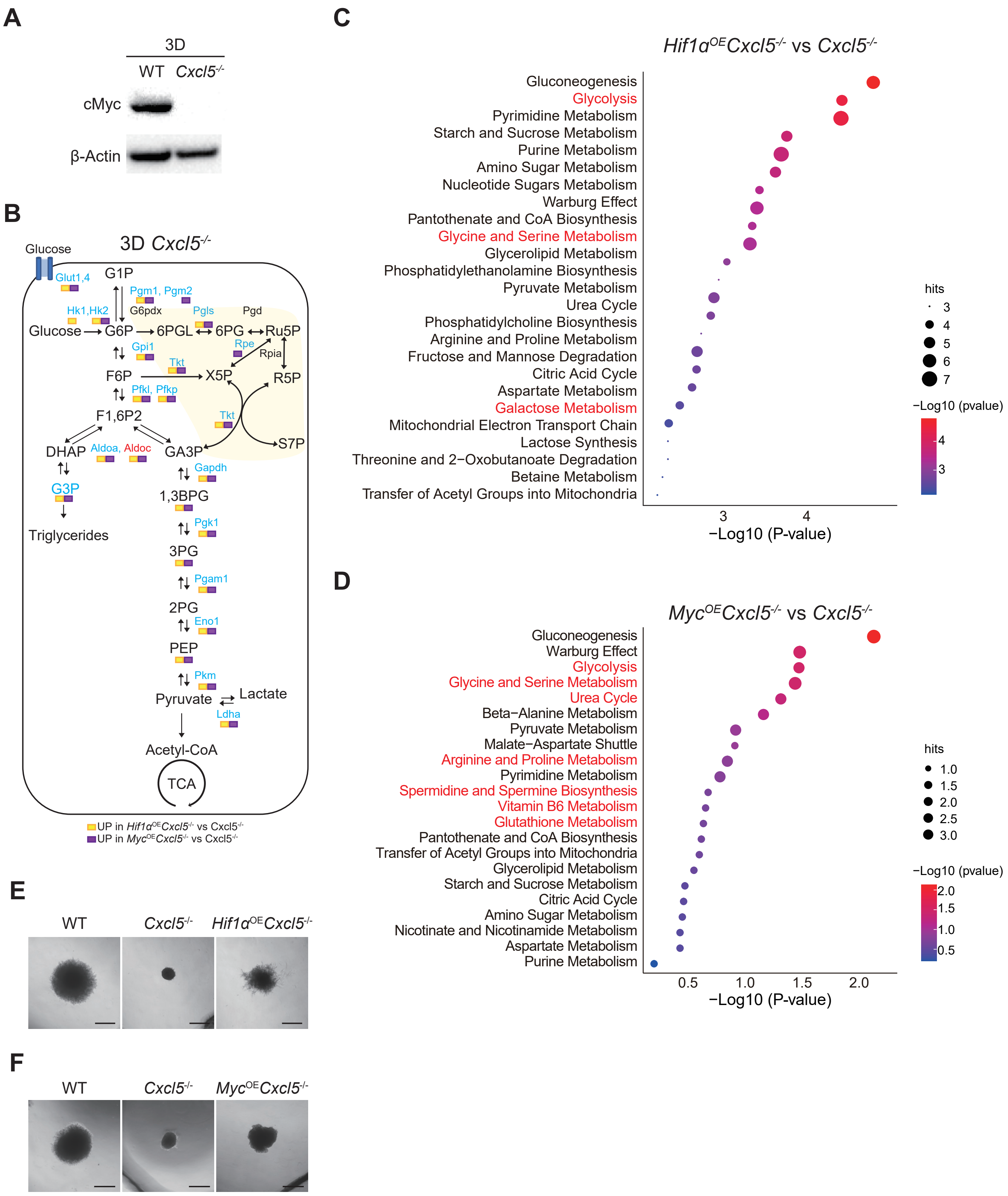

Supplementary Fig 4. *Hif1α* or *Myc* overexpression rescues the impaired metabolism and growth of 3D-cultured *Cxcl5*$^{-/-}$ cells.

A, Western blot analysis of cMyc expression in 3D WT and *Cxcl5*$^{-/-}$ cells. β-Actin, loading control (n = 3). **B**, Schematic showing the glycolysis-related pathway genes restored by *Hif1α* (brown) or *Myc* (purple) overexpression in 3D-cultured *Cxcl5*$^{-/-}$ cells. **C**, **D**, Metabolomics showing the metabolic pathways positively enriched in *Hif1α*-overexpressing (**C**) or *Myc*-overexpressing *Cxcl5*$^{-/-}$ cells (**D**) compared to parental *Cxcl5*$^{-/-}$ cells. **E**, **F**, Bright field micrographs of 3D-cultured WT, *Cxcl5*$^{-/-}$, and *Hifα*^{OE}*Cxcl5*$^{-/-}$ cells (**E**) or *Myc*^{OE}*Cxcl5*$^{-/-}$ cells (**F**). Magnification, 4X. Scale bar = 500 μm. Data are representative of three independent experiments.

Supplemantary Fig 5.

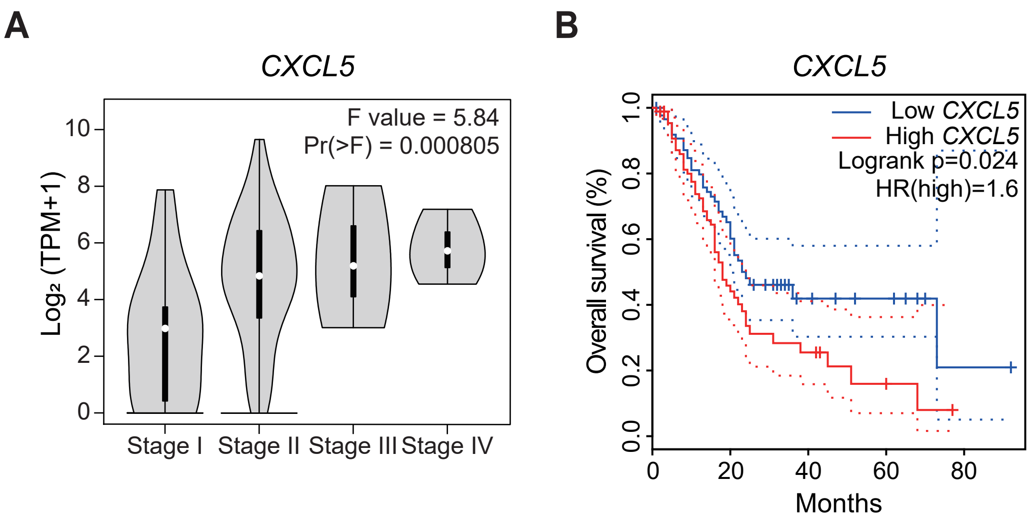

Supplementary Fig 5. CXCL5 upregulation correlates with poor PAAD patient survival.

A, In log2-normalized TCGA/GEPIA data (TPM+1), CXCL5 expression increases with increasing PAAD pathological stage. **B**, From the same TCGA/GEPIA dataset, CXCL5 expression is inversely correlated overall PAAD patient survival.

Supplementary Fig 6.

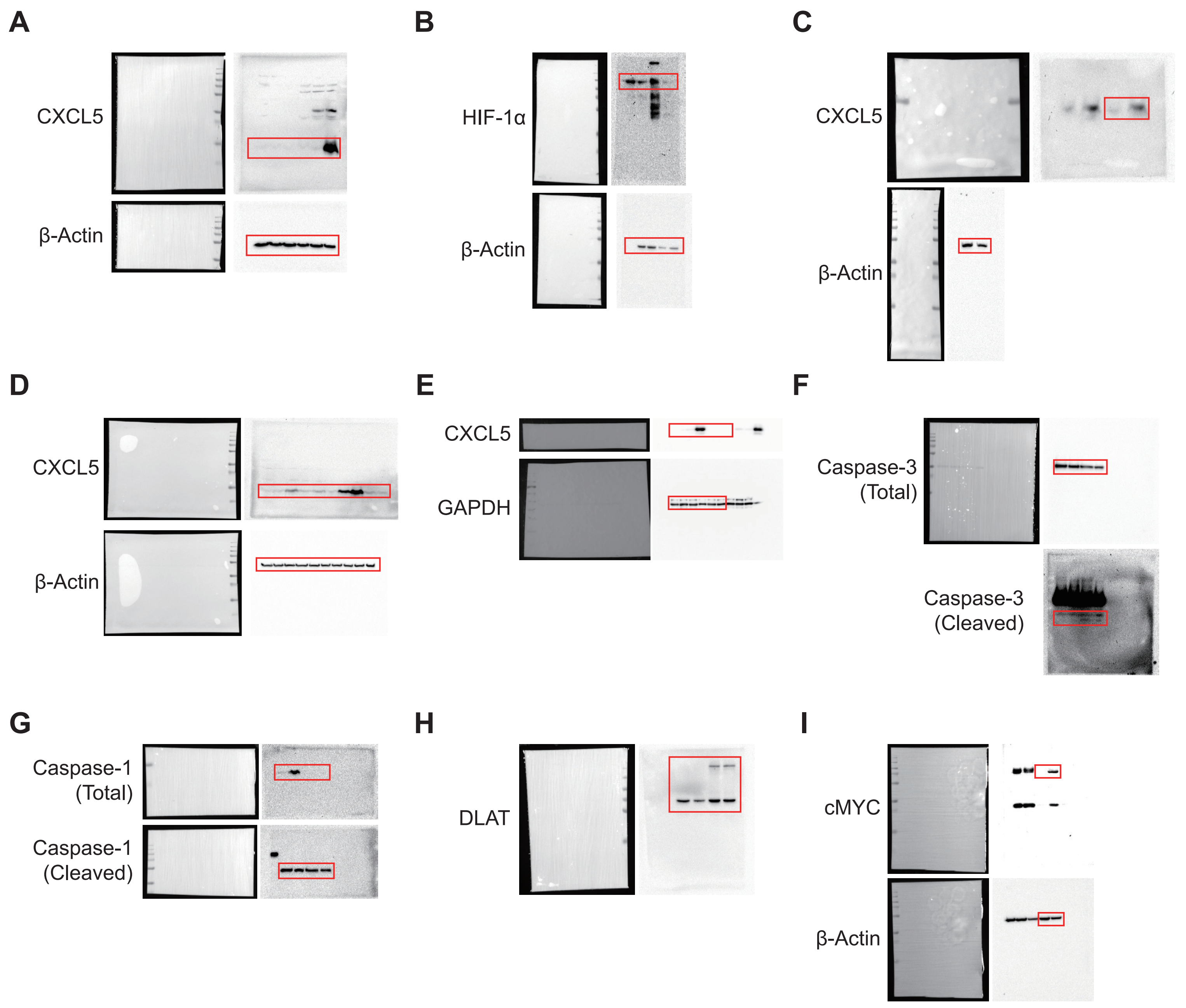

Supplementary Fig 6. Uncropped western blots.

A-I, Uncropped western blots used for panel Figure 1I (**A**), Figure 3C (**B**), Supplementary Fig. 1H (**C**), Supplementary Fig. 1J (**D**), Supplementary Fig. 2A (**E**), Supplementary Fig. 3A (**F**), Supplementary Fig. 3B (**G**), Supplementary Fig. 3C (**H**), and Supplementary Fig. 4A (**I**).

Table S1. Primers used in this study

Table S2. Up- or down-regulated genes in M1-CM treated 3D-cultured cancer cells compared to control or M0-CM treated cells

Table S3. Changes in level of metabolites

Table S4. CXCL5-correlated genes in PAAD patients
