# Supplementary material for: Cancer-intrinsic Cxcl5 orchestrates a global metabolic reprogramming for resistance to oxidative cell death in 3D
Source: Cell Death Differ. 2025 Mar 7;32(7):1200–13. doi: 10.1038/s41418-025-01466-y (PMC12284152; doi:10.1038/s41418-025-01466-y)
Supplement: Supplementary file 2 — Table S1 [file 41418_2025_1466_MOESM2_ESM.docx]

|  |  | Forward | Reverse |
| --- | --- | --- | --- |
| RT-qPCR | *Actb* | TCCAGCCTTCCTTCTTGGGT | GCACTGTGTTGGCATAGAGGT |
|  | *iNOS* | GTTCTCAGCCCAACAATACAAGA | GTGGACGGGTCGATGTCAC |
|  | *Cxcl5* | GTTCCATCTCGCCATTCATGC | GCGGCTATGACTGAGGAAGG |
|  | *Hif1α* | CAGAGCAGGAAAGAGAGTCATAGAAC | TTTCGCTT CCTCTGAGCATTC |
|  | *Myc* | GCGACTCTGAAGAAGAGCAAG | GCCTCGGGATGGAGATGAG |

Table S1.
