# Supplementary material for: Cancer-intrinsic Cxcl5 orchestrates a global metabolic reprogramming for resistance to oxidative cell death in 3D
Source: Cell Death Differ. 2025 Mar 7;32(7):1200–13. doi: 10.1038/s41418-025-01466-y (PMC12284152; doi:10.1038/s41418-025-01466-y)
Supplement: Supplementary file 3 — Table S2 [file 41418_2025_1466_MOESM3_ESM.docx]

**Table S2.**

| **Upregulated genes** | |  |  |
| --- | --- | --- | --- |
| M1 vs control | | | |
| gene_symbol | log2FC | pvalue | padj |
| C3 | 4.107624 | 2.03E-38 | 4.20E-34 |
| Cxcl5 | 2.752334 | 8.28E-36 | 8.50E-32 |
| Saa3 | 10.29719 | 6.45E-16 | 4.40E-12 |
| Cfb | 2.555916 | 3.69E-14 | 1.90E-10 |
| Slc16a2 | 4.256785 | 3.33E-13 | 1.40E-09 |
| Osbp2 | 20.88026 | 4.83E-13 | 1.70E-09 |
| Slc6a12 | 2.370925 | 4.32E-12 | 1.30E-08 |
| Elf3 | 9.136407 | 4.59E-11 | 1.20E-07 |
| Lcn2 | 9.968779 | 4.99E-09 | 1.00E-05 |
| Col9a3 | 8.365372 | 6.32E-09 | 1.20E-05 |
| Dram1 | 1.796126 | 1.67E-08 | 2.90E-05 |
| Slpi | 1.571913 | 1.99E-08 | 3.10E-05 |
| Gm13963 | 8.153304 | 4.56E-08 | 6.70E-05 |
| Prl2c2 | 1.628879 | 1.08E-07 | 1.30E-04 |
| Ifitm1 | 1.65246 | 1.06E-07 | 1.30E-04 |
| Edil3 | 8.192056 | 1.22E-07 | 1.40E-04 |
| Cfap54 | 8.13527 | 2.56E-07 | 2.80E-04 |
| Sema6c | 7.915516 | 3.50E-07 | 3.60E-04 |
| Fbln2 | 2.815556 | 4.67E-07 | 4.40E-04 |
| Mmp9 | 2.281664 | 7.75E-07 | 6.90E-04 |
| Ighm | 2.050763 | 8.99E-07 | 7.70E-04 |
| Cdh17 | 7.673368 | 1.15E-06 | 8.40E-04 |
| Jakmip3 | 7.722769 | 1.11E-06 | 8.40E-04 |
| Il1rap | 1.888081 | 1.20E-06 | 8.50E-04 |
| Tnip3 | 2.494716 | 1.41E-06 | 9.10E-04 |
| Klra2 | 2.758344 | 1.42E-06 | 9.10E-04 |
| Gm5970 | 6.620215 | 1.37E-06 | 9.10E-04 |
| Cxcl1 | 7.78432 | 1.52E-06 | 9.40E-04 |
| H2-DMb2 | 7.615342 | 2.54E-06 | 1.50E-03 |
| Ifitm3 | 1.157339 | 3.74E-06 | 2.10E-03 |
| Psmb9 | 1.353727 | 4.65E-06 | 2.50E-03 |
| Gm49936 | 8.180309 | 4.65E-06 | 2.50E-03 |
| Lbp | 3.283299 | 5.37E-06 | 2.80E-03 |
| Ppm1h | 1.976687 | 5.70E-06 | 2.90E-03 |
| Gm30489 | 7.69614 | 7.38E-06 | 3.50E-03 |
| Hdac11 | 7.301684 | 1.28E-05 | 5.80E-03 |
| 1110008P14Rik | 1.232512 | 1.32E-05 | 5.90E-03 |
| Oas1g | 2.879747 | 1.52E-05 | 6.60E-03 |
| Cck | 7.410032 | 1.76E-05 | 7.30E-03 |
| Lag3 | 7.341503 | 1.78E-05 | 7.30E-03 |
| Myb | 1.48452 | 2.21E-05 | 8.50E-03 |
| Prkcg | 7.262831 | 2.49E-05 | 9.30E-03 |
| Gm16685 | 2.475818 | 2.51E-05 | 9.30E-03 |
| Bcl3 | 1.565928 | 3.03E-05 | 1.10E-02 |
| 7420700N18Rik | 7.668102 | 3.51E-05 | 1.20E-02 |
| Enpp2 | 2.150631 | 3.82E-05 | 1.30E-02 |
| Ltbp2 | 1.906484 | 4.28E-05 | 1.40E-02 |
| Ndor1 | 2.025976 | 5.67E-05 | 1.80E-02 |
| Atp8b4 | 1.682683 | 6.31E-05 | 2.00E-02 |
| D030040B21Rik | 7.209232 | 6.57E-05 | 2.00E-02 |
| Gm50237 | 7.395406 | 6.79E-05 | 2.00E-02 |
| Adamts7 | 1.1526 | 8.60E-05 | 2.60E-02 |
| Lrrc32 | 7.22591 | 9.08E-05 | 2.60E-02 |
| Hp | 3.864414 | 9.09E-05 | 2.60E-02 |
| Gm45660 | 2.672343 | 9.41E-05 | 2.70E-02 |
| Il2rg | 5.933173 | 0.000139 | 3.80E-02 |
| Prl2c3 | 1.589328 | 0.000148 | 3.90E-02 |
| 1810044D09Rik | 6.88824 | 0.000148 | 3.90E-02 |
| Il33 | 1.526642 | 0.000152 | 4.00E-02 |
| Gm9824 | 1.722363 | 0.000153 | 4.00E-02 |
| Gm22513 | 1.488 | 0.000162 | 4.10E-02 |
| C1rb | 1.032625 | 0.000169 | 4.30E-02 |
| Gm26444 | 1.193596 | 0.000189 | 4.70E-02 |

| M1 vs M0 | | | |
| --- | --- | --- | --- |
| gene_symbol | log2FC | pvalue | padj |
| Cxcl5 | 2.553249 | 4.29E-65 | 8.80E-61 |
| Lcn2 | 7.10616 | 1.41E-56 | 1.50E-52 |
| C3 | 3.482648 | 1.84E-19 | 1.30E-15 |
| Commd1b | 10.77615 | 7.30E-15 | 3.70E-11 |
| Osbp2 | 20.80133 | 1.80E-14 | 6.10E-11 |
| Cfb | 3.062922 | 1.65E-14 | 6.10E-11 |
| Saa3 | 6.334204 | 5.01E-14 | 1.50E-10 |
| Dram1 | 1.982532 | 1.72E-13 | 4.40E-10 |
| 1110008P14Rik | 1.380529 | 6.42E-13 | 1.50E-09 |
| Slc16a2 | 3.803552 | 4.59E-12 | 9.40E-09 |
| Hp | 2.919085 | 1.91E-11 | 3.30E-08 |
| Slpi | 1.26474 | 8.83E-09 | 1.30E-05 |
| Fbln2 | 3.791142 | 2.00E-08 | 2.60E-05 |
| Enpp2 | 2.731875 | 7.52E-08 | 8.50E-05 |
| Slc39a4 | 8.034097 | 1.02E-07 | 1.10E-04 |
| Vcam1 | 1.504708 | 2.94E-07 | 3.00E-04 |
| Ifitm1 | 1.227202 | 7.43E-07 | 7.20E-04 |
| Atp8b4 | 1.873889 | 9.13E-07 | 8.50E-04 |
| Ltbp2 | 2.172167 | 1.90E-06 | 1.70E-03 |
| Lbp | 2.235751 | 6.08E-06 | 5.20E-03 |
| Gm38055 | 4.007806 | 1.90E-05 | 1.60E-02 |
| Myh13 | 1.088294 | 2.31E-05 | 1.80E-02 |
| Cebpd | 1.008661 | 5.31E-05 | 3.60E-02 |

| M0 vs Ctrl | | | |
| --- | --- | --- | --- |
| gene_symbol | log2FC | pvalue | padj |
| Gm4673 | 3.95285 | 1.19E-31 | 2.80E-27 |
| Rps26-ps1 | 1.846499 | 1.79E-21 | 2.10E-17 |
| Ighm | 2.428711 | 1.15E-16 | 9.00E-13 |
| Zfp91 | 9.858657 | 1.69E-13 | 9.90E-10 |
| Prl2c2 | 1.291932 | 2.06E-09 | 9.60E-06 |
| Gm49936 | 9.051962 | 1.56E-07 | 5.10E-04 |
| Edil3 | 8.130293 | 1.76E-07 | 5.10E-04 |
| H2-DMb2 | 7.890109 | 2.33E-07 | 6.10E-04 |
| Col9a3 | 7.856486 | 2.61E-07 | 6.10E-04 |
| Jakmip3 | 7.863538 | 3.01E-07 | 6.40E-04 |
| Itih2 | 7.615109 | 1.18E-06 | 2.30E-03 |
| Prl2c3 | 1.384185 | 2.46E-06 | 3.80E-03 |
| Avil | 7.557374 | 2.19E-06 | 3.80E-03 |
| Abca13 | 7.103398 | 3.60E-05 | 4.20E-02 |
| Astl | 5.424187 | 3.84E-05 | 4.30E-02 |
| Arhgap4 | 7.208427 | 4.05E-05 | 4.30E-02 |
| Wnt11 | 7.243146 | 5.83E-05 | 4.90E-02 |
| Gm10252 | 1.785429 | 6.78E-05 | 4.90E-02 |
| Dpf1 | 1.209172 | 6.16E-05 | 4.90E-02 |
| Gm26877 | 6.952546 | 6.74E-05 | 4.90E-02 |

| **Downregulated genes** | |  |  |
| --- | --- | --- | --- |
| M1 vs control | | | |
| gene_symbol | log2FC | pvalue | padj |
| Yy2 | -8.37131 | 4.74E-07 | 4.40E-04 |
| Gm44503 | -7.64837 | 6.51E-05 | 2.00E-02 |
| Tafa5 | -7.15995 | 1.88E-05 | 7.60E-03 |
| Atp2a3 | -7.07483 | 2.79E-05 | 1.00E-02 |
| Rpl27a-ps4 | -2.57436 | 7.40E-06 | 3.50E-03 |
| Fam167a | -2.394 | 1.48E-10 | 3.40E-07 |
| Grik2 | -2.28168 | 1.63E-05 | 7.00E-03 |
| Acox2 | -1.64976 | 1.80E-06 | 1.10E-03 |
| Lmo7 | -1.38506 | 9.11E-08 | 1.20E-04 |
| Plin4 | -1.19314 | 7.11E-06 | 3.50E-03 |
| Ass1 | -1.1617 | 6.47E-05 | 2.00E-02 |
| Gcnt1 | -1.1319 | 1.97E-05 | 7.80E-03 |
| 1700023H06Rik | -1.04964 | 1.02E-05 | 4.70E-03 |
| Pparg | -1.04439 | 1.08E-06 | 8.40E-04 |

| M1 vs M0 | | | |
| --- | --- | --- | --- |
| gene_symbol | log2FC | pvalue | padj |
| Gm12992 | -10.0179 | 8.25E-12 | 1.53E-08 |
| Yy2 | -8.63969 | 7.30E-09 | 1.15E-05 |
| Abca13 | -6.8534 | 5.04E-05 | 0.035537 |
| Snhg4 | -3.50988 | 5.26E-08 | 6.33E-05 |
| Car6 | -2.99416 | 4.68E-05 | 0.034194 |
| Rps26-ps1 | -1.39591 | 1.80E-08 | 2.45E-05 |
| Ramp1 | -1.37577 | 2.02E-05 | 0.015893 |
| Vwa1 | -1.03513 | 7.25E-05 | 0.046325 |

| M0 vs Ctrl | | | |
| --- | --- | --- | --- |
| gene_symbol | log2FC | pvalue | padj |
| Gm14819 | -8.49177 | 2.71E-09 | 1.10E-05 |
| Gm49721 | -5.10651 | 2.93E-05 | 3.80E-02 |
| Cntf | -2.08291 | 2.43E-06 | 3.80E-03 |
| Col27a1 | -1.34543 | 5.64E-05 | 4.90E-02 |
| Pcdhb9 | -1.31434 | 8.37E-06 | 1.20E-02 |
| Fam167a | -1.17094 | 3.60E-05 | 4.20E-02 |
