# Supplementary material for: Cancer-intrinsic Cxcl5 orchestrates a global metabolic reprogramming for resistance to oxidative cell death in 3D
Source: Cell Death Differ. 2025 Mar 7;32(7):1200–13. doi: 10.1038/s41418-025-01466-y (PMC12284152; doi:10.1038/s41418-025-01466-y)
Supplement: Supplementary file 4 — Table S3 [file 41418_2025_1466_MOESM4_ESM.docx]

**Table S3**.

Changes in level of metabolites between 3D WT and 2D WT

| **3D WT vs 2D WT** |  |  |  |  |
| --- | --- | --- | --- | --- |
| ID | log2FC | p.value | -Log10(p.value) | Diff |
| Phosphocreatine | 2.9299 | 0.040066 | 1.3972 | UP |
| Hypotaurine | 2.833 | 0.003887 | 2.4104 | UP |
| D-gluconate | 2.8176 | 0.018273 | 1.7382 | UP |
| O-acetyl-L-carnitine | 2.6849 | 0.059429 | 1.226 | UP |
| DHAP (Dihydroxyacetone phosphate) | 2.522 | 0.095813 | 1.0186 | UP |
| GA3P (D-glyceraldehyde 3-phosphate) | 2.522 | 0.095813 | 1.0186 | UP |
| Taurine | 2.4461 | 0.001161 | 2.9353 | UP |
| N-carbamoyl-L-aspartate | 2.4461 | 0.047384 | 1.3244 | UP |
| citraconic acid | 2.3453 | 0.002044 | 2.6895 | UP |
| 2-Hydroxyglutarate | 2.1212 | 1.95E-05 | 4.71 | UP |
| Pyroglutamate | 2.0994 | 0.000799 | 3.0973 | UP |
| Creatinine | 2.083 | 5.95E-05 | 4.2251 | UP |
| L-Cystine | 2.0424 | 0.002806 | 2.5519 | UP |
| Allantoin | 1.8848 | 0.00219 | 2.6595 | UP |
| Carnosine | 1.832 | 0.000708 | 3.1502 | UP |
| sn-Glycero-3-phosphocholine | 1.8174 | 0.000117 | 3.9301 | UP |
| ATP (Adenosine 5'-triphosphate) | 1.786 | 0.06142 | 1.2117 | UP |
| N-acetyl-L-carnitine | 1.6943 | 0.000456 | 3.3413 | UP |
| N-acetyl-L-cysteine | 1.6821 | 0.000699 | 3.1554 | UP |
| G3P (Glycerol 3-phosphate) | 1.5775 | 0.015957 | 1.797 | UP |
| Choline (pos, net +1) | 1.4523 | 9.05E-05 | 4.0432 | UP |
| Pyridoxine | 1.4472 | 0.004944 | 2.306 | UP |
| D-Glucosamine 6-phosphate | 1.1929 | 0.032565 | 1.4872 | UP |
| Acetylcholine (pos, net +1) | 1.189 | 0.015112 | 1.8207 | UP |
| Malate | 1.1842 | 0.001148 | 2.9401 | UP |
| Pantothenate | 0.98103 | 0.00215 | 2.6676 | UP |
| IMP (Inosine 5'-phosphate) | 0.97251 | 0.025319 | 1.5966 | UP |
| UDP-N-acetyl-D-galactosamine | 0.51098 | 0.057363 | 1.2414 | UP |
| Deoxyuridine | -4.7768 | 0.023183 | 1.6348 | DOWN |
| 4-Aminobutyrate | -2.625 | 0.000591 | 3.2288 | DOWN |
| Cytosine | -2.3789 | 0.00072 | 3.1424 | DOWN |
| Uracil | -1.698 | 0.041471 | 1.3823 | DOWN |
| L-Asparagine | -1.4771 | 0.004742 | 2.324 | DOWN |
| cAMP (3',5'-cyclic AMP) | -1.3025 | 0.028556 | 1.5443 | DOWN |
| Ornithine | -1.2572 | 0.007783 | 2.1089 | DOWN |
| Uridine 5'-monophosphate (UMP) | -1.2053 | 0.051103 | 1.2916 | DOWN |
| Cytidine 5'-diphosphate (CDP) | -1.1825 | 0.011134 | 1.9534 | DOWN |
| GSH (Glutathione) | -1.1665 | 0.0021 | 2.6779 | DOWN |
| SAH (S-adenosyl-L-homocysteine) | -0.99075 | 0.090116 | 1.0452 | DOWN |
| L-Histidine (pos) | -0.97375 | 0.003622 | 2.4411 | DOWN |
| NADPH (Nicotinamide Adenine Dinucleotide Phosphate Reduced) | -0.95785 | 0.0954 | 1.0205 | DOWN |
| UDP-D-glucuronate | -0.92256 | 0.008254 | 2.0833 | DOWN |
| L-Glutamate | -0.90586 | 0.014215 | 1.8472 | DOWN |
| Ribose 1-phosphate | -0.8865 | 0.092071 | 1.0359 | DOWN |
| GSSG (Glutathione disulfide) | -0.76089 | 0.05027 | 1.2987 | DOWN |
| L-Cystathionine | -0.74758 | 0.033114 | 1.48 | DOWN |
| Methionine | -0.67865 | 0.057973 | 1.2368 | DOWN |
| GDP (Guanosine 5'-diphosphate) | -0.58059 | 0.047288 | 1.3253 | DOWN |
| S-methyl-5'-thioadenosine | -0.57416 | 0.022901 | 1.6401 | DOWN |
| N,3-Bisphospho-D-glycerate | 5.2978 | 0.21464 | 0.6683 | NO |
| PEP (Phosphoenolpyruvate) | 2.7262 | 0.21914 | 0.65927 | NO |
| 3PG (3-phosphoglycerate) | 2.5814 | 0.16108 | 0.79297 | NO |
| Uridine 5'-triphosphate (UTP) | 2.2533 | 0.11279 | 0.94774 | NO |
| 3-Phosphoserine | -1.7591 | 0.2976 | 0.52637 | NO |
| Cytidine-5'-monophosphate (CMP) | -1.6878 | 0.12137 | 0.91589 | NO |
| N-Carbamoyl-beta-alanine | -1.3883 | 0.18051 | 0.74349 | NO |
| AMP (Adenosine 5'-phosphate) | -1.3822 | 0.13139 | 0.88145 | NO |
| GMP (Guanosine 5'-phosphate) | -1.1409 | 0.23249 | 0.6336 | NO |
| GTP (Guanosine 5'-triphosphate) | 0.99422 | 0.31144 | 0.50662 | NO |
| Cytidine 5'-triphosphate (CTP) | 0.88548 | 0.29393 | 0.53175 | NO |
| N-Acetyl-L-methionine | -0.84599 | 0.28815 | 0.54038 | NO |
| N-Acetyl-L-glutamate | -0.7081 | 0.28779 | 0.54092 | NO |
| Acetyl phosphate | 0.67202 | 0.13287 | 0.87657 | NO |
| N-Acetyl-L-leucine | 0.66745 | 0.22824 | 0.64161 | NO |
| Uric acid | -0.65728 | 0.15232 | 0.81723 | NO |
| PRPP (5-phosphoribosyl 1-pyrophosphate) | 0.63497 | 0.23492 | 0.62909 | NO |
| NAD+ (Nicotinamide Adenine Dinucleotide)2 | -0.59821 | 0.21548 | 0.6666 | NO |
| Guanosine | -0.56492 | 0.46379 | 0.33368 | NO |
| L-Argininosuccinate | -0.52615 | 0.10776 | 0.96756 | NO |
| 2-Keto-D-gluconic acid | 0.51665 | 0.20802 | 0.68189 | NO |
| Creatine | -0.49476 | 0.000438 | 3.3589 | NO |
| L-Tyrosine | -0.46774 | 0.43972 | 0.35682 | NO |
| NADP+ (Nicotinamide Adenine Dinucleotide Phosphate) | -0.45073 | 0.3413 | 0.46686 | NO |
| L-Citrulline | -0.42137 | 0.30002 | 0.52285 | NO |
| Inosine | 0.40575 | 0.65377 | 0.18457 | NO |
| Xanthine | 0.37606 | 0.51803 | 0.28565 | NO |
| Tryptophan | -0.3502 | 0.018449 | 1.734 | NO |
| Choline phosphate (pos, net +1) | 0.33233 | 0.064613 | 1.1897 | NO |
| Uridine 5'-diphosphate (UDP) | 0.31437 | 0.4776 | 0.32093 | NO |
| cis-Aconitate | 0.31082 | 0.54271 | 0.26543 | NO |
| Thiamine (pos, net +1) | 0.27389 | 0.19847 | 0.70231 | NO |
| L-Phenylalanine | -0.26825 | 0.018279 | 1.738 | NO |
| beta-NMN (beta-Nicotinamide mononucleotide) | -0.25475 | 0.69341 | 0.15901 | NO |
| 4-Pyridoxate | 0.25287 | 0.55284 | 0.2574 | NO |
| L-Isoleucine | -0.24576 | 0.064387 | 1.1912 | NO |
| N-acetyl-L-asparagine | -0.24222 | 0.4066 | 0.39083 | NO |
| L-Alanine | 0.2371 | 0.47509 | 0.32322 | NO |
| Hydroxyproline | -0.21882 | 0.71966 | 0.14287 | NO |
| Carnitine (1) (pos) | -0.21647 | 0.50798 | 0.29416 | NO |
| Homocysteine | 0.20098 | 0.72068 | 0.14226 | NO |
| Adenosine | 0.18828 | 0.74403 | 0.12841 | NO |
| ADP (Adenosine 5'-diphosphate) | -0.18268 | 0.50399 | 0.29758 | NO |
| SAM (S-Adenosylmethionine) | 0.17192 | 0.12409 | 0.90627 | NO |
| L-Glutamine | -0.1595 | 0.82795 | 0.081995 | NO |
| Citrate | 0.15771 | 0.74778 | 0.12623 | NO |
| Aspartate | -0.15585 | 0.55703 | 0.25412 | NO |
| L-Proline | -0.10523 | 0.30996 | 0.5087 | NO |
| Adenine | -0.0899 | 0.59859 | 0.22287 | NO |
| Betaine (pos, 118) | 0.08378 | 0.70039 | 0.15466 | NO |
| 2-oxoglutarate | 0.075362 | 0.89555 | 0.047911 | NO |
| Cytidine 5-diphosphocholine | 0.074416 | 0.77393 | 0.1113 | NO |
| NADH (Nicotinamide Adenine Dinucleotide Reduced) | 0.055169 | 0.88546 | 0.052833 | NO |
| L-Homoserine | -0.01955 | 0.90906 | 0.041407 | NO |
| N-Acetyl-D-mannosamine | 0.01914 | 0.94314 | 0.025423 | NO |
| L-Kynurenine | 0.002813 | 0.97166 | 0.012484 | NO |
| N-Acetyl-L-aspartate | -0.00266 | 0.99442 | 0.0024308 | NO |

Changes in level of metabolites between 3D Cxcl5^-/-^ and 3D WT

| **3D *Cxcl5^-/-^* vs 3D WT** |  |  |  |  |
| --- | --- | --- | --- | --- |
| ID | log2FC | p.value | -Log10(p.value) | Diff |
| N-carbamoyl-L-aspartate | -7.0602 | 0.025024 | 1.6016 | DOWN |
| Phosphocreatine | -5.4415 | 0.027585 | 1.5593 | DOWN |
| Cytidine 5'-triphosphate (CTP) | -5.4003 | 0.05077 | 1.2944 | DOWN |
| NADH (Nicotinamide Adenine Dinucleotide Reduced) | -5.2077 | 0.001461 | 2.8353 | DOWN |
| IMP (Inosine 5'-phosphate) | -4.1251 | 0.002407 | 2.6186 | DOWN |
| Cytidine 5'-diphosphate (CDP) | -4.0134 | 0.024949 | 1.6029 | DOWN |
| ATP (Adenosine 5'-triphosphate) | -3.8526 | 0.027877 | 1.5548 | DOWN |
| cAMP (3',5'-cyclic AMP) | -3.4081 | 0.000169 | 3.7719 | DOWN |
| Uridine 5'-triphosphate (UTP) | -3.1946 | 0.082171 | 1.0853 | DOWN |
| Adenosine | -3.1165 | 0.030471 | 1.5161 | DOWN |
| Inosine | -3.0799 | 0.035669 | 1.4477 | DOWN |
| citraconic acid | -2.8808 | 0.000761 | 3.1185 | DOWN |
| Choline phosphate (pos, net +1) | -2.6963 | 1.79E-05 | 4.746 | DOWN |
| 2-Hydroxyglutarate | -2.653 | 2.35E-05 | 4.6281 | DOWN |
| 2-oxoglutarate | -2.5913 | 0.002384 | 2.6227 | DOWN |
| NAD+ (Nicotinamide Adenine Dinucleotide)2 | -2.4464 | 0.020304 | 1.6924 | DOWN |
| L-Citrulline | -2.3926 | 0.042843 | 1.3681 | DOWN |
| PRPP (5-phosphoribosyl 1-pyrophosphate) | -2.2717 | 0.034786 | 1.4586 | DOWN |
| Pantothenate | -2.2395 | 0.000375 | 3.4259 | DOWN |
| ADP (Adenosine 5'-diphosphate) | -2.2218 | 0.014896 | 1.8269 | DOWN |
| NADPH (Nicotinamide Adenine Dinucleotide Phosphate Reduced) | -2.1341 | 0.004387 | 2.3578 | DOWN |
| UDP-D-glucuronate | -1.8473 | 0.009343 | 2.0295 | DOWN |
| Ornithine | -1.7443 | 0.000148 | 3.8292 | DOWN |
| L-Proline | -1.7324 | 1.45E-05 | 4.8382 | DOWN |
| Pyroglutamate | -1.6604 | 0.001837 | 2.7359 | DOWN |
| Malate | -1.617 | 0.000749 | 3.1256 | DOWN |
| L-Cystathionine | -1.5833 | 0.002858 | 2.544 | DOWN |
| GSSG (Glutathione disulfide) | -1.4393 | 0.002045 | 2.6893 | DOWN |
| GSH (Glutathione) | -1.4177 | 0.0132 | 1.8794 | DOWN |
| Cytidine 5-diphosphocholine | -1.4054 | 0.021469 | 1.6682 | DOWN |
| Adenine | -1.3571 | 0.000439 | 3.3578 | DOWN |
| GDP (Guanosine 5'-diphosphate) | -1.2492 | 0.036199 | 1.4413 | DOWN |
| L-Glutamate | -1.1479 | 0.011205 | 1.9506 | DOWN |
| UDP-N-acetyl-D-galactosamine | -1.0904 | 0.009063 | 2.0427 | DOWN |
| G3P (Glycerol 3-phosphate) | -1.009 | 0.046884 | 1.329 | DOWN |
| L-Kynurenine | -0.99674 | 0.000261 | 3.5841 | DOWN |
| S-methyl-5'-thioadenosine | -0.92162 | 0.008259 | 2.0831 | DOWN |
| Uridine 5'-diphosphate (UDP) | -0.82746 | 0.088025 | 1.0554 | DOWN |
| sn-Glycero-3-phosphocholine | -0.81019 | 0.001804 | 2.7437 | DOWN |
| L-Alanine | -0.77432 | 0.019019 | 1.7208 | DOWN |
| Creatinine | -0.7733 | 0.001903 | 2.7205 | DOWN |
| NADP+ (Nicotinamide Adenine Dinucleotide Phosphate) | -0.72732 | 0.060631 | 1.2173 | DOWN |
| Thiamine (pos, net +1) | -0.66357 | 0.011154 | 1.9526 | DOWN |
| L-Phenylalanine | -0.53102 | 0.002927 | 2.5336 | DOWN |
| Guanosine | -3.6621 | 0.14923 | 0.82613 | NO |
| N,3-Bisphospho-D-glycerate | -3.568 | 0.24141 | 0.61724 | NO |
| GTP (Guanosine 5'-triphosphate) | -2.6687 | 0.10588 | 0.97518 | NO |
| 3-Phosphoserine | -2.5324 | 0.18769 | 0.72655 | NO |
| AMP (Adenosine 5'-phosphate) | -2.0493 | 0.19814 | 0.70304 | NO |
| Uracil | -1.6778 | 0.12759 | 0.8942 | NO |
| O-acetyl-L-carnitine | -1.677 | 0.10044 | 0.99811 | NO |
| Cytidine-5'-monophosphate (CMP) | -1.4527 | 0.10849 | 0.96459 | NO |
| PEP (Phosphoenolpyruvate) | -1.4181 | 0.35278 | 0.4525 | NO |
| 3PG (3-phosphoglycerate) | -1.254 | 0.3067 | 0.51328 | NO |
| DHAP (Dihydroxyacetone phosphate) | -1.2385 | 0.22704 | 0.6439 | NO |
| GA3P (D-glyceraldehyde 3-phosphate) | -1.2385 | 0.22704 | 0.6439 | NO |
| GMP (Guanosine 5'-phosphate) | -1.1205 | 0.25338 | 0.59623 | NO |
| Cytosine | 0.99297 | 0.15682 | 0.80461 | NO |
| Hydroxyproline | -0.92179 | 0.12316 | 0.90952 | NO |
| Ribose 1-phosphate | 0.72611 | 0.28175 | 0.55013 | NO |
| cis-Aconitate | 0.67634 | 0.14069 | 0.85175 | NO |
| N-acetyl-L-asparagine | -0.58513 | 0.15208 | 0.81793 | NO |
| Aspartate | -0.42493 | 0.031417 | 1.5028 | NO |
| L-Homoserine | -0.42039 | 0.004242 | 2.3725 | NO |
| 2-Keto-D-gluconic acid | 0.39972 | 0.010373 | 1.9841 | NO |
| Homocysteine | -0.37555 | 0.50692 | 0.29506 | NO |
| Tryptophan | -0.3699 | 0.029128 | 1.5357 | NO |
| beta-NMN (beta-Nicotinamide mononucleotide) | 0.36747 | 0.52788 | 0.27747 | NO |
| Taurine | 0.34412 | 0.096346 | 1.0162 | NO |
| Hypotaurine | -0.33592 | 0.24016 | 0.61949 | NO |
| Citrate | 0.33365 | 0.32515 | 0.48791 | NO |
| SAH (S-adenosyl-L-homocysteine) | -0.29849 | 0.6147 | 0.21134 | NO |
| Allantoin | -0.26614 | 0.30507 | 0.5156 | NO |
| Xanthine | 0.26474 | 0.81301 | 0.089905 | NO |
| N-acetyl-L-carnitine | 0.2598 | 0.021217 | 1.6733 | NO |
| L-Asparagine | 0.24966 | 0.52167 | 0.28261 | NO |
| L-Argininosuccinate | -0.23682 | 0.55482 | 0.25585 | NO |
| L-Isoleucine | -0.21092 | 0.028819 | 1.5403 | NO |
| Uridine 5'-monophosphate (UMP) | -0.15069 | 0.80595 | 0.093693 | NO |
| Pyridoxine | 0.097505 | 0.70529 | 0.15163 | NO |
| L-Tyrosine | 0.095165 | 0.80092 | 0.096411 | NO |
| Methionine | -0.08714 | 0.28284 | 0.54846 | NO |
| SAM (S-Adenosylmethionine) | 0.057924 | 0.52426 | 0.28045 | NO |
| Acetyl phosphate | -0.05753 | 0.85098 | 0.070083 | NO |
| Deoxyuridine | -0.01662 | 0.98118 | 0.0082524 | NO |
| N-Acetyl-L-glutamate | -0.0125 | 0.97449 | 0.011223 | NO |
| Carnosine | 0.012474 | 0.96269 | 0.016515 | NO |
| Betaine (pos, 118) | 2.6402 | 0.000147 | 3.8319 | UP |
| N-Acetyl-L-leucine | 2.3162 | 0.005314 | 2.2745 | UP |
| Acetylcholine (pos, net +1) | 2.0474 | 4.15E-05 | 4.3819 | UP |
| 4-Aminobutyrate | 1.4297 | 0.004099 | 2.3873 | UP |
| N-Acetyl-L-methionine | 1.3772 | 0.002038 | 2.6908 | UP |
| N-Acetyl-D-mannosamine | 1.2278 | 0.022008 | 1.6574 | UP |
| 4-Pyridoxate | 1.2167 | 0.01269 | 1.8966 | UP |
| D-Glucosamine 6-phosphate | 1.1613 | 0.061929 | 1.2081 | UP |
| N-acetyl-L-cysteine | 1.0552 | 0.018823 | 1.7253 | UP |
| D-gluconate | 1.0509 | 0.033064 | 1.4807 | UP |
| Uric acid | 1.0389 | 0.027116 | 1.5668 | UP |
| L-Cystine | 0.92585 | 0.01076 | 1.9682 | UP |
| N-Acetyl-L-aspartate | 0.86231 | 0.023036 | 1.6376 | UP |
| N-Carbamoyl-beta-alanine | 0.85811 | 0.085762 | 1.0667 | UP |
| Carnitine (1) (pos) | 0.7908 | 0.096149 | 1.0171 | UP |
| Serine | 0.77409 | 0.001595 | 2.7972 | UP |
| L-Glutamine | 0.7345 | 0.008069 | 2.0932 | UP |
| Choline (pos, net +1) | 0.67358 | 0.001656 | 2.7809 | UP |
| Creatine | 0.64008 | 0.00012 | 3.9209 | UP |
| L-Histidine (pos) | 0.50314 | 0.014235 | 1.8466 | UP |

Rescued metabolites by overexpressing Hif1*α* or Myc in Cxcl5^-/-^ cells.

|  | ***Cxcl5^-/-^* vs WT** | **vs *Cxcl5^-/-^*** | | **vs WT** | |
| --- | --- | --- | --- | --- | --- |
| **Metabolite** |  | ***Myc^OE^***  ***Cxcl5^-/-^*** | ***Hif1α^OE^***  ***Cxcl5^-/-^*** | ***Myc^OE^***  ***Cxcl5^-/-^*** | ***Hifα^OE^***  ***Cxcl5^-/-^*** |
| N-Acetyl-L-aspartate | ↑ | ↓ | ↓ | - | - |
| N-Acetyl-L-leucine | ↑ | ↓ | ↓ | - | - |
| D-gluconate | ↑ | ↓ | ↓ | ↓ | ↓ |
| N-acetyl-L-carnitine | ↑ | ↓ | ↓ | ↓ | ↓ |
| Creatine | ↑ | ↓ | ↓ | ↓ | - |
| Uridine 5'-diphosphate (UDP) | ↓ | ↓ | ↓ | ↓ | ↓ |
| NADP+ (Nicotinamide Adenine Dinucleotide Phosphate) | ↓ | ↓ | ↓ | ↓ | ↓ |
| N,3-Bisphospho-D-glycerate | - | ↑ | ↑ | ↑ | - |
| PEP (Phosphoenolpyruvate) | - | ↑ | ↑ | ↑ | - |
| Uracil | - | ↑ | ↑ | ↑ | - |
| 3-Phosphoserine | - | ↑ | ↑ | ↑ | ↑ |
| Cytosine | - | ↑ | - | ↑ | ↑ |
| Pyridoxine | - | ↑ | - | ↑ | - |
| Deoxyuridine | - | ↑ | - | ↑ | - |
| Malate | ↓ | ↑ | - | - | ↓ |
| Thiamine (pos, net +1) | ↓ | ↑ | - | - | ↓ |
| L-Phenylalanine | ↓ | ↑ | - | - | ↓ |
| Pyroglutamate | ↓ | ↑ | - | ↓ | ↓ |
| 2-Hydroxyglutarate | ↓ | ↑ | - | ↓ | ↓ |
| L-Citrulline | ↓ | ↑ | ↑ | - | ↓ |
| Pantothenate | ↓ | ↑ | ↑ | - | ↓ |
| Ornithine | ↓ | ↑ | ↑ | - | ↓ |
| Choline phosphate (pos, net +1) | ↓ | ↑ | ↑ | ↓ | ↓ |
| NADPH (Nicotinamide Adenine Dinucleotide Phosphate Reduced) | ↓ | ↓ | - | ↓ | ↓ |
| UDP-D-glucuronate | ↓ | ↓ | - | ↓ | ↓ |
| GSSG (Glutathione disulfide) | ↓ | ↓ | - | ↓ | ↓ |
| GSH (Glutathione) | ↓ | ↓ | - | ↓ | ↓ |
| Cytidine 5-diphosphocholine | ↓ | ↓ | - | ↓ | ↓ |
| Adenine | ↓ | ↓ | - | ↓ | ↓ |
| GDP (Guanosine 5'-diphosphate) | ↓ | ↓ | - | ↓ | ↓ |
| UDP-N-acetyl-D-galactosamine | ↓ | ↓ | - | ↓ | ↓ |
| Acetylcholine (pos, net +1) | ↑ | ↓ | - | ↓ | ↑ |
| L-Glutamine | ↑ | ↓ | - | ↑ | ↑ |
| G3P (Glycerol 3-phosphate) | ↓ | ↑ | ↑ | - | ↑ |
| NADH (Nicotinamide Adenine Dinucleotide Reduced) | ↓ | x | ↑ | ↓ | ↓ |
| N-carbamoyl-L-aspartate | ↓ | x | ↑ | ↓ | ↓ |
| Phosphocreatine | ↓ | - | ↑ | ↓ | ↓ |
| IMP (Inosine 5'-phosphate) | ↓ | - | ↑ | ↓ | ↓ |
| Cytidine 5'-diphosphate (CDP) | ↓ | - | ↑ | ↓ | ↓ |
| cAMP (3',5'-cyclic AMP) | ↓ | - | ↑ | ↓ | ↓ |
| Adenosine | ↓ | - | ↑ | ↓ | ↓ |
| L-Cystathionine | ↓ | - | ↑ | ↓ | ↓ |
| Cytidine 5'-triphosphate (CTP) | ↓ | - | ↑ | ↓ | - |
| ATP (Adenosine 5'-triphosphate) | ↓ | - | ↑ | ↓ | - |
| sn-Glycero-3-phosphocholine | ↓ | - | ↑ | ↓ | - |
| Uridine 5'-triphosphate (UTP) | ↓ | ↓ | ↑ | ↓ | - |
| NAD+ (Nicotinamide Adenine Dinucleotide)2 | ↓ | ↓ | ↑ | ↓ | ↓ |
| ADP (Adenosine 5'-diphosphate) | ↓ | ↓ | ↑ | ↓ | ↓ |
| Betaine (pos, 118) | ↑ | - | ↓ | ↑ | - |
| 4-Aminobutyrate | ↑ | - | ↓ | ↑ | - |
| N-acetyl-L-cysteine | ↑ | - | ↓ | ↑ | - |
| Serine | ↑ | - | ↓ | ↑ | - |
| Uric acid | ↑ | - | ↓ | ↑ | - |
| L-Histidine (pos) | ↑ | - | ↓ | ↑ | - |
| N-Carbamoyl-beta-alanine | ↑ | - | ↓ | - | - |
| 2-Keto-D-gluconic acid | ↑ | - | ↓ | - | ↓ |
| L-Cystine | ↑ | - | ↓ | - | ↓ |
| Carnitine (1) (pos) | ↑ | - | ↓ | ↑ | ↓ |
| Choline (pos, net +1) | ↑ | - | ↓ | ↑ | ↑ |
| N-Acetyl-L-methionine | ↑ | ↓ | ↓ | - | ↓ |
| 4-Pyridoxate | ↑ | ↑ | ↓ | ↑ | - |
| N-Acetyl-D-mannosamine | ↑ | ↑ | ↓ | ↑ | ↓ |
| L-Proline | ↓ | - | ↓ | - | ↓ |
| Creatinine | ↓ | - | ↓ | ↓ | ↓ |
| L-Homoserine | ↓ | - | ↓ | ↓ | ↓ |
| L-Glutamate | ↓ | - | - | ↓ | ↓ |
| Inosine | ↓ | - | - | ↓ | ↓ |
| citraconic acid | ↓ | - | - | ↓ | ↓ |
| 2-oxoglutarate | ↓ | - | - | ↓ | ↓ |
| PRPP (5-phosphoribosyl 1-pyrophosphate) | ↓ | - | - | ↓ | ↓ |
| L-Kynurenine | ↓ | - | - | ↓ | ↓ |
| S-methyl-5'-thioadenosine | ↓ | - | - | ↓ | ↓ |
