# Supplementary material for: Cancer-intrinsic Cxcl5 orchestrates a global metabolic reprogramming for resistance to oxidative cell death in 3D
Source: Cell Death Differ. 2025 Mar 7;32(7):1200–13. doi: 10.1038/s41418-025-01466-y (PMC12284152; doi:10.1038/s41418-025-01466-y)
Supplement: Supplementary file 5 — Table S4 [file 41418_2025_1466_MOESM5_ESM.docx]

**Table S4**.

CXCL5-correlated genes associated with hypoxia and metabolism

| Hypoxia | Glycolysis-associated metabolism | Monosaccharide metabolism | Nitrogen metabolism | Sterol biosynthesis |
| --- | --- | --- | --- | --- |
| CA9 | CTH | PGM2 | CTH | HMGCS1 |
| CSRP2 | DCN | PFKFB3 | CA9 | INSIG2 |
| ERO1A | ENO1 | GALE | CA12 | MSMO1 |
| GJA1 | ERO1A |  |  | SQLE |
| GPRC5A | GALE |  |  |  |
| INSIG2 | HMMR |  |  |  |
| ISG20 | IER3 |  |  |  |
| KLF6 | ISG20 |  |  |  |
| KLF7 | KDELR3 |  |  |  |
| LOXL2 | KIF20A |  |  |  |
| MET | LDHA |  |  |  |
| PFKFB3 | MET |  |  |  |
| PLAUR | PFKFB3 |  |  |  |
| SAT1 | PGM2 |  |  |  |
| SLCO4A1 | PPP2CA |  |  |  |
| STC1 | SLC35A3 |  |  |  |
| TIPARP | STC1 |  |  |  |
| ANLN | GSTO1 |  |  |  |
| CA12 |  |  |  |  |
| CNIH4 |  |  |  |  |
| LDHA |  |  |  |  |
| LDLR |  |  |  |  |
| S100A3 |  |  |  |  |
| SLC16A1 |  |  |  |  |
| ENO1 |  |  |  |  |
| HMOX1 |  |  |  |  |
| ACKR3 |  |  |  |  |
| ANXA2 |  |  |  |  |
| DCN |  |  |  |  |
| IER3 |  |  |  |  |
| KDELR3 |  |  |  |  |
| PGM2 |  |  |  |  |
| TNFAIP3 |  |  |  |  |
| HDAC9 |  |  |  |  |
| HIF1A |  |  |  |  |
